# Penile implants and other high risk practices in French Guiana’s correctional facility: A cause for concern

**DOI:** 10.1371/journal.pone.0218992

**Published:** 2019-06-28

**Authors:** Marie-Claire Parriault, Amandine Chaponnay, Claire Cropet, Vincent About, Agathe Pastre, Roch Perusseau-Lambert, Mathieu Nacher, Florence Huber

**Affiliations:** 1 Centre d’Investigation Clinique Antilles Guyane, CIC INSERM, Cayenne General Hospital, Cayenne, French Guiana, France; 2 COREVIH, Cayenne General Hospital, Cayenne, French Guiana, France; 3 UCSA, Cayenne General Hospital, Cayenne, French Guiana, France; 4 Association Guyanaise de Réduction des Risques, Cayenne, French Guiana, France; 5 University of French Guiana, Cayenne, French Guiana, France; 6 Réseau Kikiwi, Cayenne, French Guiana, France; University of South Florida, UNITED STATES

## Abstract

**Background:**

Prisoners in French Guiana, a French territory located in South America, have a HIV and hepatitis B prevalence of 4%. Body modifications such as penile implants, tattoos, and body piercings are common among detainees, increasing the risk of blood-borne virus transmission.

**Methods:**

We conducted a cross-sectional randomised survey in which the primary objective was to estimate the prevalence of high risk ‘bloody practices’ (penile implants, tattoos, body piercings) in French Guiana’s only correctional facility. The secondary objective was to describe the risk factors for penile implants, the procedures and motivations for insertion, the reported complications, their risk factors and adverse impact on condom use.

**Results:**

Of the 221 male inmates interviewed, 19% had tattoos or body piercings while incarcerated, and 68% had penile implants, of which, 85% had been inserted inside the correctional facility. Addictive behaviors such as cannabis use and alcohol addiction (positive AUDIT-C score), early age at first sexual intercourse, and the number of incarcerations correlated positively with having inserted one or more penile implants while incarcerated. In contrast, having reported previous psychiatric hospitalizations and having a high knowledge score for HIV/AIDS and sexually transmitted infections (STIs) were negatively correlated with the insertion of penile implants while incarcerated. Penile implants were inserted in poor hygienic conditions, usually using the sharp lid of a canned food container, with 18% of early complications, mostly haemorrhage and edema. Condom use was negatively impacted for 52% of men with penile implants.

**Conclusions:**

Our results highlight the need for prevention interventions which should aim at increasing knowledge levels and at implementing comprehensive risk-reduction measures.

## Introduction

Inmate populations are particularly affected by sexually transmitted infections (STIs) and the Human Immunodeficiency Virus (HIV) with a reported prevalence between three to six times higher than in the general population [1,2]. French Guiana, a French overseas territory located in South America, has the highest incarceration rate among French territories and in Latin America [3]. HIV prevalence among inmates in French Guiana was 3.9% [4], and chronic hepatitis B (HBV) with positive Hbs antigen was 4.1%, none were infected with hepatitis C (HCV) [5]. Intravenous drug use is not a common practice in French Guiana and thus viral transmission occurs primarily through sexual contact [6]. However, some practices observed in correctional facilities can increase the risk of transmission, especially for HBV, mainly through body modifications such as tattoos, body piercings and penile implants, which are usually called « *bouglous* » in French Guiana.

One of the first reference on penile implants was made in the Vatsyayana Kama Sutra which describes various body modifications consisting of inserting stimulating objects under the skin of the penis [7,8]. Since then, this practice has been widely described around the world; in Asia, especially among the Yakuza in Japan [9] and practiced by southeast Asian men [10,11]; in Slavic populations; and occasionally, among men living in Western Europe and America [8,12,13,14,15]. These different populations shared one common feature: most implants were inserted during or following a prison stay [13,15,16]. The link between incarceration and high risk “bloody practices” has previously been reported for penile implants, for tattoos and for body piercings [16,17].

In correctional facilities, tattoos, body piercings and penile implants are often performed under poor hygienic conditions, with inappropriate equipment, thus increasing the risk of HIV, hepatitis B and hepatitis C transmission [16,18,19,20]. Reusing and sharing tattoo needles have been reported [20]. Incisions have been made with spoons or toothbrushes that are sharpened against a wall, a concrete slab or the floor. Instruments are often cleaned prior to their use, but not always [19].

Penile implants are widespread among prisoners in French Guiana. Of 492 newly incarcerated men who took part in a cross-sectional survey, 29.6% reported having a penile implant [21,22].

To understand this phenomenon in more detail, we conducted another cross-sectional survey among men incarcerated in French Guiana. The primary objective was to estimate the prevalence of penile implants, tattoos, and body piercings, collectively described as “bloody practices”, in French Guiana’s correctional facility. A secondary objective was to describe the procedures and motivations for insertion, the associated medical complications, the impact on condom use, and the risk of blood borne virus transmission. This study was under taken to provide guidance on prevention measures and ultimately to reduce the burden of medical complications among prisoners in French Guiana’s correctional facility.

## Methods

### Ethical and regulatory approval

This study was approved by the French Regulatory authorities CNIL (*Commission Nationale Informatique et Libertés*, authorization no. 1840401v0) and by the Ethical Committee of INSERM (*Institut National de la Santé et de la Recherche Médicale*, IRB000038888, avis no. 15–207). Participants were fully informed and gave written consent to participate in the study. No identifier variables were retained in the study database after randomization.

### Study population

The study, conducted between March 31^st^ 2015 and July 1^st^2015, randomly selected inmates aged 18 years and above, who were incarcerated in the French Guiana correctional facility on the date of March 4^th^ 2015. The correctional facility consisted of: a prison for men sentenced two years or more *(‘centre de détention’);* and a separate jail for men and women who were awaiting trial or sentenced for a duration of less than two years *(‘maison d’arrêt’)*. The total incarcerated population was 729 detainees on 1 January 2014, while the initial correctional facility capacity was 614 detainees [23,24].

### Study questionnaire

A structured questionnaire of 140 questions was used. The design of the questionnaire was based on several Knowledge, Attitudes, Beliefs and Practices studies (KABP) conducted in the general population and vulnerable populations such as sex workers, crack cocaine users, men who have sex with men and migrants living in French Guiana or mainland France [25,26,27,28,29]. The questions from the KABP studies were adapted to the local inmate population. The questionnaire was translated by one certified and four qualified language teachers into five languages (French, English, Spanish, Portuguese and Sranan Tongo). The main languages spoken in French Guiana are French and Creole. The translated questionnaires were then tested and validated by peer health facilitators who spoke at least one of these languages. Health facilitators were from non-government organizations working with the incarcerated populations; they were also interviewers and were trained in survey techniques and questionnaire administration. The questions mainly explored sociodemographic characteristics, life in the correctional facility, sexual history and experience, alcohol and drug use.

### Sampling method and study conduct

**Fig 1:**
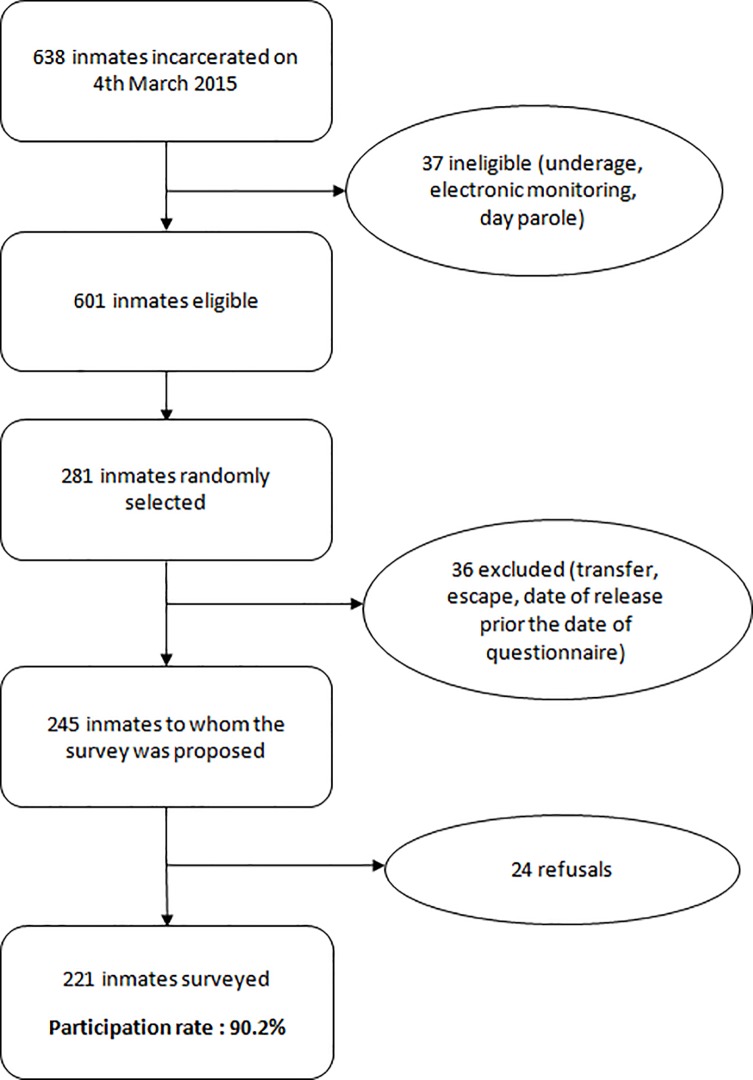
Recruitment of male inmates.

A cross-sectional random sampling was used to initially select inmates for the face to face survey in the correctional facility. If they agreed to participate in the survey, a schedule was organized based on the timing of interviewers’ visits and the corresponding languages they spoke. There were five interviewers for this survey. The recruitment and participants’ selection process is illustrated in more detail in Fig 1.

### Statistical analysis

Only the results concerning male inmates are presented here. A descriptive analysis of the variables was performed using means and standard deviations for normally distributed continuous variables, medians and interquartile ranges for non-normally distributed continuous variables, and frequencies and percentages for qualitative variables.

Bivariate and multivariate modified Poisson regressions [30] were then performed, and prevalence ratios were used to identify factors associated with having declared at least one penile implant, and with having declared at least one complication of penile implants. The odds ratios in analyses of cross-sectional studies with binary outcomes can significantly overestimate the relative risk notably when the association is strong and/or when the event is frequent, thus in our situation prevalence ratios are the measure of choice. Modified Poisson regression provides correct estimates and is a better alternative for this type of analysis [30]. Given the large number of variables and the potential spurious associations, explanatory variables were first selected according to a literature review of the local context, then variables with a *p<0*.*20* in the bivariate analyses were retained in the final multivariate model. Variance inflation factors were used to check for collinearity. The residual deviance was used to perform goodness-of-fit for each selected model. Analyses were conducted with Stata 13.0 (StataCorp, College Station, TX).

The Alcohol Use Disorders Identification Test (AUDIT-C) was part of the questionnaire. It is a validated 3-item alcohol screening method that can help identify patients who are hazardous drinkers or have active alcohol use disorders (including alcohol abuse or dependence) [31]. The AUDIT-C is scored on a scale of 0–12. A score of 4 or more is considered positive for men.

The level of certain knowledge of inmates was evaluated with 8 questions concerning knowledge of HIV/AIDS and STIs, modes of transmission and prevention. Each correct answer was scored with 1 point (‘does not know’, ‘no answer’ and a wrong answer was 0). The highest score was 8 points, the worst was 0. This score is not a validated score.

## Results

### Sociodemographic and detention characteristics

A total of 221 male inmates were surveyed, representing 34.6% of the total male prison population in French Guiana, with a 90.2% response rate (Fig 1). Sociodemographic characteristics of male inmates are shown Table 1.

**Table 1:** Inmates’ sociodemographic characteristics.

	Number	%
*Age (n = 221)*		
18–24	57	25.8
25–34	84	38.0
35–44	43	19.5
45 and more	37	16.7
*Country of birth (n = 220)*		
France	89	40.4
Suriname	43	19.6
Brazil	29	13.2
Guyana	40	18.2
Other	19	8.6
Age when school was interrupted (n = 207)*		
	Mean	16.8 (+/- 3.3)
	Min : 8	Max : 29
*Involved in a relationship (n = 218)*		
Yes	128	58.7
No	90	41.3
*Total length of incarceration (n = 218)*		
0–11 months	70	32.1
12–35 months	76	34.9
36 months and more	72	33.0
*Number of incarcerations (n = 221)*		
First incarceration	115	52.0
One or two past incarcerations	58	26.3
Three past incarcerations and more	48	21.7

*14 inmates had never been to school

### Addictive behaviors

The Alcohol Use Disorders Identification Test (AUDIT-C) was positive for 57% of inmates interviewed. The score was 4.36 (+/-3.44) among male inmates surveyed. Cannabis consumption was common, 60.6% of persons reported using cannabis every day or more than once per week before or during the incarceration. None used cocaine, crack-cocaine or blaka (crack and cannabis mixed and smoked together) during incarceration. Pre-incarceration, 5.9% used crack, 2.3% cocaine and 4.5% blaka every day or more than once per week.

Among inmates interviewed, 10.8% had previously been hospitalized in a psychiatric unit while 39% had been followed-up by the outpatient psychiatric unit (UFPI).

### Sexuality and sexual risk behaviors

Most men reported they were heterosexual (95.4%). Over the last five years, 64.1% reported having had several sexual partners during the same period and 36.7% reported having had sex with commercial sex partners. Over the past 12 months, men reported having had 1.9 (+/-3.2) sexual partners. Those incarcerated for less than a year, on average, had 2.96 partners (+/- 3.60) over the past 12 months, while those incarcerated for over a year had 0.64 partners (+/- 1.54) on average over the past 12 months. The median age at the first intercourse was 14 years. Among those surveyed, 29.8% had previously been diagnosed with an STI. The mean knowledge score was 4.4 (+/-1.4), the minimum score was 1 and the maximum was 8.

### Tattoos, body piercings and penile implants

More than two-thirds of men surveyed (67%, n = 149) had one or more penile implants, and 19% (n = 41) reported they had been given tattoos or body piercings while incarcerated.

Among those who had a penile implant, more than half (85%, n = 127) inserted them during incarceration, with 6.7 (+/- 6.5) penile implants on average [range 1–50 penile implants]. Half (53%) had five or more penile implants and 21.6% had over 9 penile implants.

Only 16% reported inserting penile implants on their own. The others received assistance from another inmate, mostly for free (76%). For 87%, the material used for placing penile implants was a sharp lid of a canned food container (usually tinned sardines sold inside the correctional facility).

For 54% of the concerned men, the main motivation for inserting penile implant was to enhance the pleasure of their sexual partners while 15% had inserted penile implants “just to try”, 9% because they found it beautiful, and 9% to enhance their own sexual pleasure.

**Fig 2:**
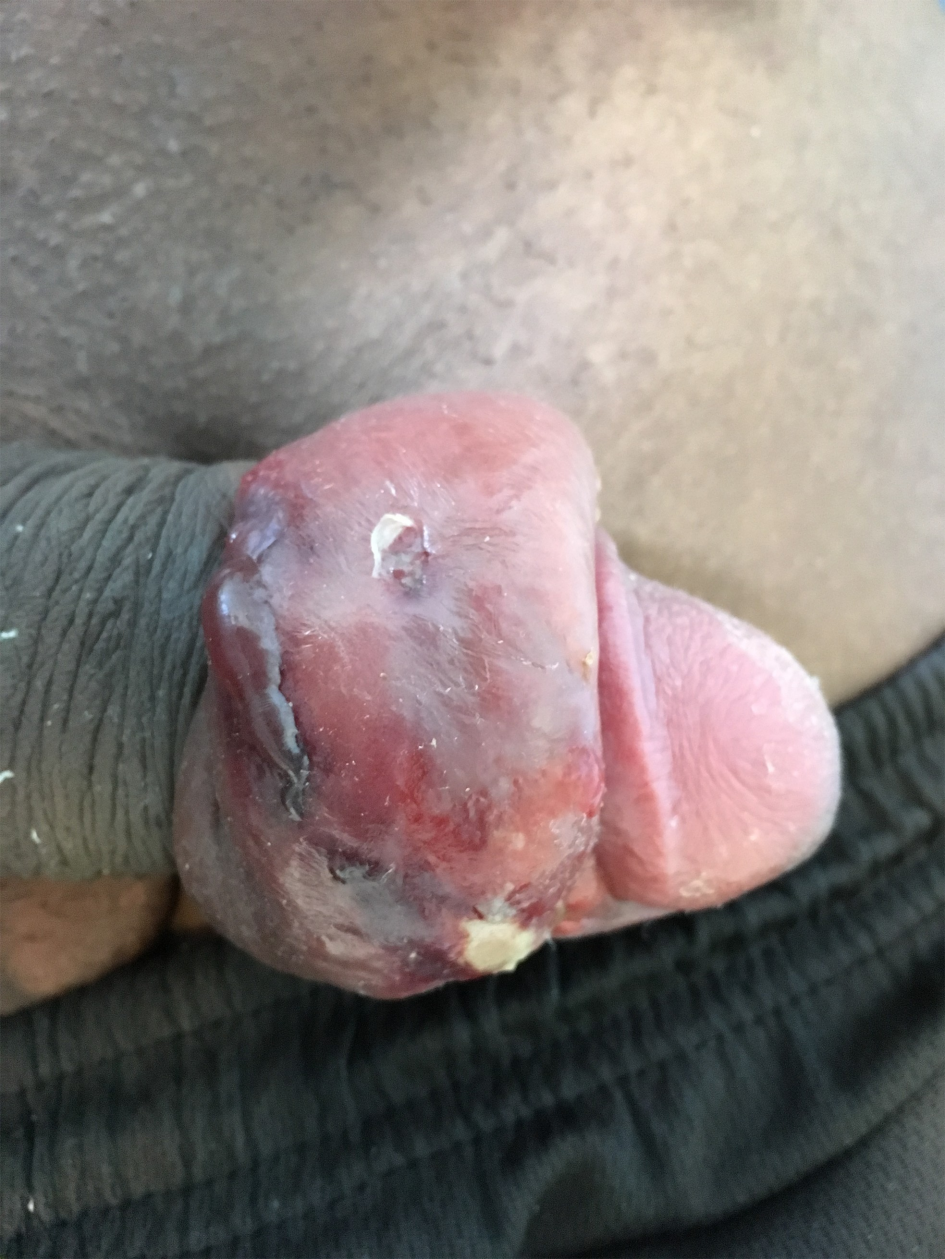
Short-term complication (infectious paraphimosis), following a penile implant insertion, in the French Guiana correctional facility.

Among men who inserted penile implants during incarceration, 18% reported one or more early complications (Fig 2) resulting in pain (n = 5), swelling after insertion (n = 15), significant bleeding (n = 6), erectile dysfunction (n = 2) and fever (n = 1).

Among other complications, penile implants interfered with the use of condoms for 52% of the men concerned (condom rupture, difficulties to insert a condom) and 36% reported using two condoms, one on top of the other, to avoid tearing and ruptures associated with the penile nodules’ physical impact on the condom.

### Multivariate analysis

Tables 2 and 3 present the bivariate analysis used to determine which variables were selected in the multivariate models.

**Table 2:** Bivariate analysis regarding penile implants inserted during incarceration.

	Penile implants inserted during incarceration	
	Crude prevalence ratios (95%CI)	*p*
*Age*		
18–24	1	***0*.*023***
25–34	1.086 (0.840–1.402)	
35–44	0.946 (0.682–1.131)	
45 and more	0.484 (0.283–0.829)	
*Country of birth*		
France	1	***0*.*055***
Surinam	1.034 (0.767–1.396)	
Brazil	0.708 (0.443–1.131)	
Guyana	1.241 (0.957–1.609)	
Other	0.630 (0.341–1.167)	
*Education*		
Has a degree	0.861 (0.680–1.091)	*0*.*216*
Does not have any degree	1	
*Involved in a relationship*		
Yes	0.854 (0.679–1.074)	***0*.*177***
No	1	
*Multiple sexual partnerships*		
Yes	1.200 (0.933–1.543)	***0*.*156***
No	1	
*History of STI*		
Yes	1.095 (0.863–1.389)	*0*.*456*
No	1	
*Psychiatric history*		
Yes	0.575 (0.324–1.019)	***0*.*058***
No	1	
*Score AUDIT-C*		
Positive	1.526 (1.180–1.973)	***0*.*001***
Negative	1	
*Cannabis use*		
Yes	2.194 (1.601–3.007)	***0*.*000***
No	1	
Knowledge of STI/HIV score*		
Score < = 4	1	
Score >4	0.765 (0.600–0.976)	***0*.*031***
*Age at first intercourse*		
< = median	1.320 (1.029–1.694)	***0*.*029***
>median	1	
*Total length of inarceration*		
0–11 months	1	***0*.*023***
12–35 months	1.412 (1.019–1.958)	
36 months and more	1.555 (1.133–2.135)	
*Number of incarceration*		
First incarceration	1	***0*.*000***
One or two past incarcerations	1.570 (1.179–2.090)	
Three and more past incarcerations	2.046 (1.600–2.617)	
*Has tatoos/body piercings*		
Yes	1.717 (1.428–2.064)	***0*.*000***
No	1	
Number of persons per cell**		
Less than 3	1	
3 or more	0.825 (0.648–1.050)	***0*.*118***

Bold values are significant<0.20

* 4 is the median score. The score ranges from 0 to 8 and is not externally validated

** 3 is the average number of persons per cell

**Table 3:** Bivariate analysis regarding declared complications after inlaying penile implant.

	With declared a complication	
	Crude prevalence ratios (95%CI)	*p*
*Age*		
18–24	1	*0*.*445*
25–34	0.540 (0.229–1.269)	
35–44	0.755 (0.287–1.988)	
45 and more	0.343 (0.048–2.435)	
*Country of birth*		
France	1	
Surinam	1.471 (0.568–3.808)	*0*.*935*
Brazil	1.062 (0.256–4.404)	
Guyana	1.319 (0.505–3.442)	
Other	0.911 (0.132–6.278)	
*Education*		
Has a degree	1.714 (0.819–3.588)	***0*.*153***
Does not have any degree	1	
*Involved in a relationship*		
Yes	0.809 (0.378–1.728)	*0*.*584*
No	1	
*Multiple sexual partnerships*		
Yes	1.036 (0.462–2.322)	*0*.*931*
No	1	
*History of STI*		
Yes	1.948 (0.939–4.040)	***0*.*073***
No	1	
*Psychiatric history*		
Yes	1.825 (0.522–6.377)	*0*.*346*
No	1	
*Score AUDIT-C*		
Positive	1.780 (0.715–4.529)	*0*.*212*
Negative	1	
*Cannabis use*		
Yes	0.847 (0.370–1.955)	*0*.*697*
No	1	
Knowledge of STI/HIV score*		
Score < = 4	1	
Score > 4	1.080 (0.506–2.307)	*0*.*841*
*Age at first intercourse*		
< = median	1.184 (0.526–2.666)	*0*.*683*
>median	1	
*Total length of incarceration*		
0–11 months	1	*0*.*577*
12–35 months	0.810 (0.338–1.945)	
36 months and more	0.604 (0.245–1.554)	
*Number of incarcerations*		
First incarceration	1	*0*.*754*
One or two past incarcerations	0.908 (0.381–2.165)	
Three and more past incarcerations	0.702 (0.278–1.773)	
*Has tattoos/body piercings*		
Yes	1.413 (0.647–3.084)	*0*.*386*
No	1	
Number of persons per cell**		
< 3	1	
> = 3	1.383 (0.647–2.958)	*0*.*403*
*Number of penile implants*		
< 10	1	
> = 10	1.868 (0.893–3.907)	***0*.*097***
*Material used for inserting penile implant*		
Razor blade	1	
Sharp lid of canned food	1.909 (0.284–12.845)	*0*.*506*
Other	1.667 (0.125–22.232)	*0*.*699*
*Helped to insert penile implant*		
Yes	1	
No	1.116 (0.423–2.945)	*0*.*825*

Bold values are significant<0.20

* 4 is the median score. The score ranges from 0 to 8 and is not externally validated

** 3 is the average number of persons per cell

Table 4 shows the factors associated with inserting penile implants during incarceration.

There were no significant factors associated with short term local complications in the multivariate model.

**Table 4:** Predictive factors for penile implants insertion in French Guiana’s correctional facility (CF).

	Inserting penile implants in CF/total (%)	Crude prevalence ratios (95% CI)	Adjusted prevalence ratios (95% CI)	p***
N = 209				
*Number of incarcerations*				
First incarceration	48/115 (41.7)	1	1	***0*.*009***
One or two past incarcerations	38/58 (65.5)	1.570 (1.179–2.090)	1.355 (1.029–1.786)	
Three and more past incarcerations	41/48 (85.4)	2.046 (1.600–2.617)	1.491 (1.153–1.927)	
*Cannabis use*				
Yes	98/134 (73.1)	2.194 (1.601–3.007)	1.737 (1.269–2.378)	***0*.*001***
No	29/87 (33.3)	1	1	
*Psychiatric history*				
Yes	8/23 (34.8)	0.575 (0.324–1.019)	0.639 (0.416–0.983)	***0*.*042***
No	115/190 (60.5)	1	1	
*Score AUDIT-C*				
Positive	85/126 (67.5)	1.526 (1.180–1.973)	1.326 (1.043–1.687)	***0*.*021***
Negative	42/95 (44.2)	1	1	
Age at first intercourse*				
< = 14 years	83/130 (63.8)	1.320 (1.029–1.694)	1.298 (1.029–1.637)	***0*.*027***
>14 years	44/91 (48.3)	1	1	
Knowledge of STI/HIV score**				
Score < = 4	80/125 (64.0)	1	1	
Score > 4	47/96 (48.9)	0.765 (0.600–0.976)	0.757 (0.612–0.938)	***0*.*011***
*Getting tatoos/body piercings in CF*				
Yes	36/41 (87.8)	1.717 (1.428–2.064)	1.222 (0.996–1.501)	*0*.*055*
No	90/176 (51.1)	1	1	

Note: Variance inflation factors values do not indicate collinearity between variables

No significant factors were associated with short term local complications in the related multivariate model

* 14 years is the median age

** 4 is the median score

*** p-values for the adjusted prevalence ratios

## Discussion

### Magnitude of the problem

This study explored the prevalence and the risk factors associated with the insertion of penile implants among inmates in French Guiana. Addictive behaviors, early age at first intercourse, and number of incarcerations correlated positively with having inserted penile implants during incarceration. Inversely, previous psychiatric hospitalizations and a high knowledge of HIV/AIDS and STI scores correlated negatively with the insertion of penile implants during incarceration.

To our knowledge, the observed proportion of male inmates declaring penile implants inserted during incarceration is the highest reported to date (68%, n = 149), higher than the reported 29.6% (43% among multiple offenders) among newly incarcerated men in French Guiana, with a significant linear trend between declaring penile implants and the number of previous incarcerations [21]. As a comparison (Table 5), prevalence was estimated at 5.8% of the total men surveyed in custody in Australia between 2006 and 2008 [16].

**Table 5:** Comparisons between cross-sectional studies of penile nodules in prison.

	Total male prisoners surveyed	Prisoners with penile implants		Prisoners with penile implants inserted while in prison		
	n	n	%	n	%	% of total surveyed
French Guiana prison study	221	149	67.4%	127	85.2%	57.5%
French Guiana newly incarcerated study (Nacher et al., 2018)	492	145	29.6%	N/A	N/A	N/A
Australian prisoners study (Yap et al., 2013)	2018	118	5.8%	87	73.7%	4.3%

### Harmful practices in a risky environment

In French Guiana, as generally reported in the literature, the majority of the penile implants were inserted during incarceration [11,16]. In prison, body modifications made in poor hygiene conditions may place the inmate at risk for blood-borne virus transmission [11,17,19,20,32,33]. According to a 2014 French Guiana correctional facility care unit report, among the 1548 inmates listed, 3% (n = 46) were Hbs Ag+ and 3.9% (n = 61) HIV-positive.

Complications from body modifications are infections and hemorrhages [8,10]. In our study, 18% of the inmates who inserted penile implants during incarceration reported edema and haemorrhage. For most of the male inmates concerned, penile implants interfered with condom use. The study found inmates had difficulties in fitting condoms over their penile nodules, and condom rupture was commonly reported leading to the superposition of condoms one on top of the other to avoid rupture.

Globally, male prisoners appeared to have riskier sexual behaviors: 29.8% reported a past history of STIs and 36.7% used the services of sex workers. Those with penile implants may be at even greater risk: sexual initiation was at an early age; and they had a lower level of HIV/STI knowledge. In Australia, having a penile implant was associated with “ever being paid for sex” in multivariate analysis [16].

Although the design of this study did not show that penile implants were associated with viral transmission, we assume that sexual intercourse with penile implants has the potential to be harmful.

**Fig 3:**
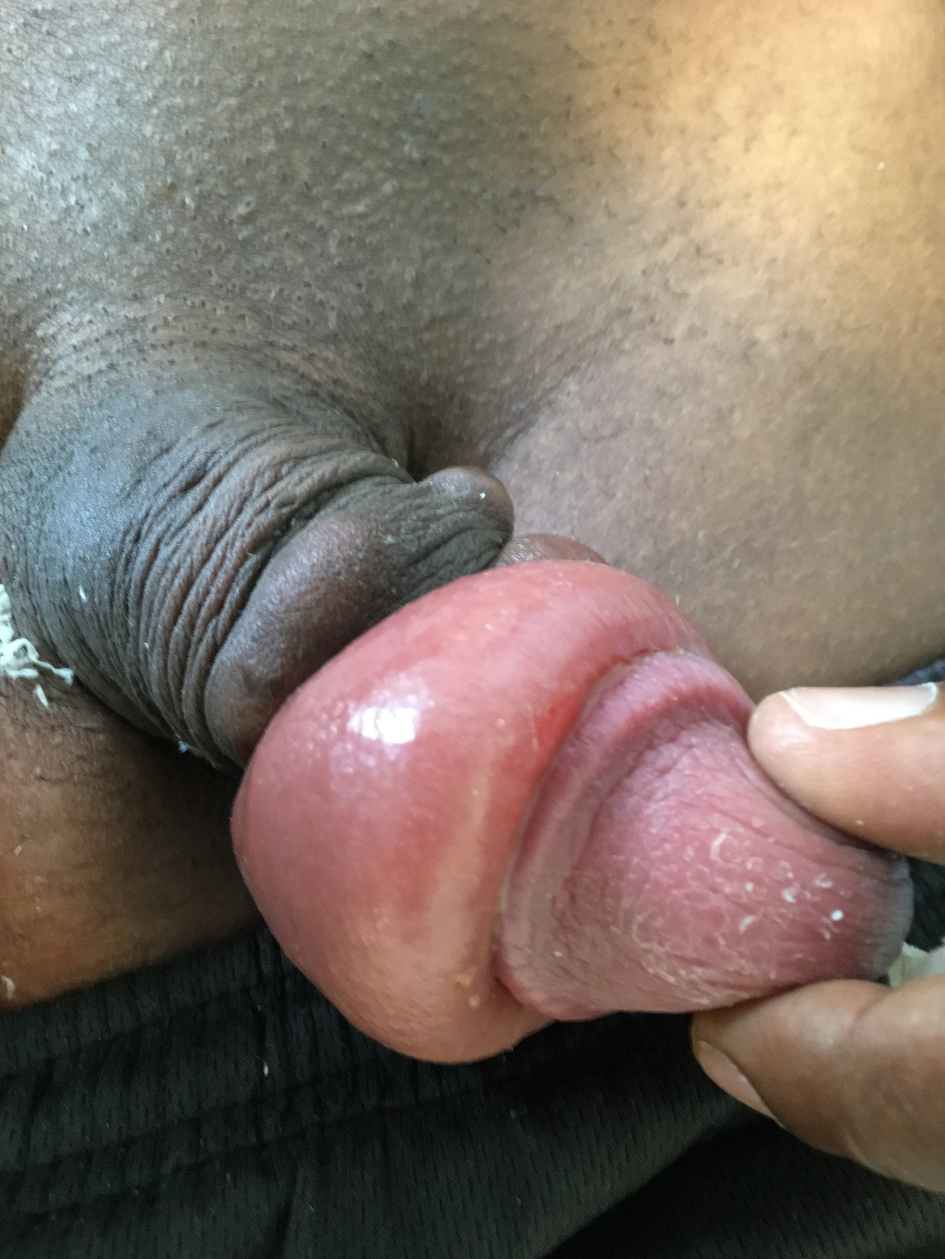
Short-term complication (infectious paraphimosis), following a penile implant insertion, in French Guiana’s correctional facility.

**Fig 4:**
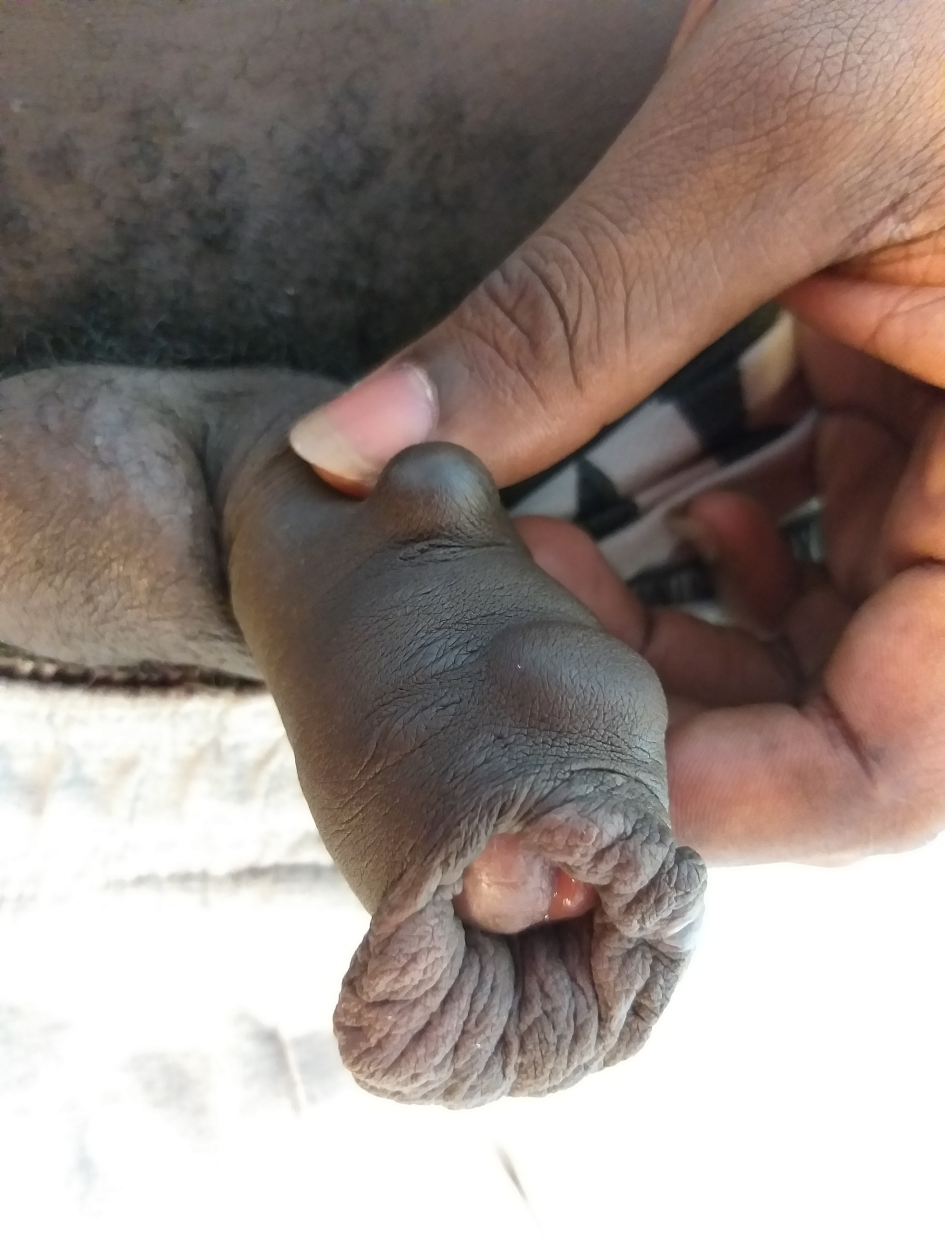
Long-term sequella, 6 months after an infectious paraphimosis following a penile implant insertion (same person than Fig 3).

In addition to the usual risks, a case report from Brazil suggested that penile implants may cause penile cancer [34]. In French Guiana, clinicians reported that individuals with penile implants experienced pain during sexual intercourse, infectious paraphimosis, a short term complication (Fig 3), and aesthetic deformity of the penis (Fig 4, six months later). Unfortunately, questions relative to late complications were not detailed in our survey.

### Penile implant and mental health

In our study, inserting penile implants while incarcerated was associated with addictive behaviors such as daily cannabis use and alcohol dependence (AUDIT-C positive score) but not cocaine consumption, which did not appear to be a risk factor. The association between penile implants and illicit drug use has already been reported in several surveys [12,16,35], and in a previous study held in French Guiana among newly incarcerated inmates [21]. We cannot explain why cocaine was not a risk factor compared to other types of addictions.

Reporting past hospitalizations for psychiatric problems seemed to be a protective factor in our setting from inserting penile implants in custody. This is consistent with results from a previous survey in French Guiana [21]. In the multivariate analysis, psychoses and suicidal risk were strongly negatively correlated with declaring penile implants among the newly incarcerated men (RR: 0.1 [0.05–0.5], p = 0.002,and RR: 0.6 [0.2–1.4], p = 0.02, respectively). The latter may be the result of social isolation and consequently, the lower influence of peer groups. The desire to enhance pleasure, the driver for the actual penile implants insertion, is presumably diminished among people who are suicidal or psychotic and who may also be taking psychotropic drugs that interfere with the dopamine pathways that are key to sexual drive. We assume that bloody practice may be linked with borderline personality disorders, frequently found among inmates [3,36]. Risky sexual behaviors, psychoactive substance use and self-mutilation are all markers frequently associated with borderline personality disorders [37,38,39]. This could be a common risk factor for these bloody practices in prison. Unfortunately, our questionnaire was not designed to test this association.

### Recidivism

As reported elsewhere [16,17,21], a strong correlation was found between having inserted penile implants during incarceration and recidivism. The length and number of previous incarcerations were strongly associated with having inserted penile implant during incarceration. The link between bloody practices and previous incarcerations, as well as, length of incarceration have already been reported [16]. These body modifications may be seen as a social behavior or as "rites" that are sensitive to peer pressure [8,11,40]. However, these practices have spread elsewhere, gaining popularity among certain groups outside of correctional facilities. In Suriname, insertion of penile implants now occurs among men living on the Maroni river banks [33]. However, there are alternative explanations regarding the relationship between recidivism and penile nodule insertions; repeat offenders may differ psychologically in their propensity to insert penile nodules, thus confounding the association. Such a latent variable that would both be associated with a greater tendency to do illegal things and to insert penile nodules would lead to what we observed: multiple incarcerations associated with greater proportions of penile nodules. In epidemiological terms the association would thus result from confounding by a third variable. Factors related to the inmates’ personality, lifestyle, and social cues may explain this relationship.

### Implications for risk reduction

Evidence showed that prevention of infectious risks is limited and not well implemented in the French correctional facility [41]. So far, there are no official recommendations regarding body modifications among inmates. These practices are rarely taken into account in national and international guidelines and deserve to be highlighted considering the extent of the phenomenon in some regions of the world, as in French Guiana.

Indeed, among incarcerated inmates who had penile implants in our study, place of birth did not appear to be a significant factor to predict which groups were more likely to practice penile implant insertions, whether they were born in Brazil, Surinam, Guyana or French Guiana. We assume that this practice maybe widespread in surrounding countries, although it is still poorly documented.

Consequently, for healthcare workers practical questions are still unresolved: what harm reduction program should be implemented? Should care givers propose or accept to remove penile implants? How can knowledge be increased among inmates regarding the risks of penile implants?

In our study, knowledge about HIV/STI was a significant protective factor in reducing penile implant insertions among male detainees. Improving the level of knowledge may be a first attempt to reduce the prevalence of penile implant insertions and may include the following information: risk of transmission of HIV, hepatitis B, STIs; practical information on infection control (sterilization of the equipment, not sharing it); women’s sexual preferences (some women are reluctant to have sex with a partner with penile implants); and condom use with penile implants [11,12,20]. Nevertheless, this information alone may not be enough for such a well entrenched practice.

Harm reduction programs should involve the prisoners. Those who decide to insert penile implants should have the opportunity to do it in hygienic conditions. In our setting, discussions are underway with a local NGO and the penitentiary administration to implement a harm reduction program (e.g. inform inmates about risks, providing sterile kits …). In other correctional facilities, a "safe" place and sterile single use material was made available to prisoners for penile implants, body piercings or tattoos [19,20]. Regular support or training by professionals (tattoo artists and body piercers), may be monitored and evaluated.

This KABP study was collected through face-to-face interviews which potentially could have impacted inmates’ answers by giving socially desirable responses to interviewers. The survey could also have been distorted by memory recall biases on answers related to past experiences and behaviors [42]. Cross-sectional surveys have some limitations; this type of study cannot prove cause and effect relationships [43]. Despite these limitations, we achieved a high response rate (90.2%) among male prisoners in this study. These results highlight the burden of bloody practices in French Guiana’s correctional facility, particularly the insertion of penile implants, which concerned over two-thirds of male inmates.

### Conclusion

In 2013, *Yap et al*. wrote an article entitled, “Penile implants among prisoners—a cause of concern?” [16]. From our French Amazonian experience, we can definitively answer that penile implants are a cause of concern in our correctional facility.

Insertion of penile implants appeared to be more common than tattoos and body piercings in the correctional facility, involving 68% of the total male inmates surveyed. The main risk factors for penile implant insertion in the correctional facility were extent of peer-to-peer social interactions, and recidivism.

These practices might increase the risk of blood-borne virus transmission and sexually transmitted infections due to the lack of infection control during insertion and problems arising from condom use post-insertion.

These results highlight the need for relevant prevention interventions that should aim to increase the level of knowledge, and set-up comprehensive risk-reduction involving the beneficiaries.

## Supporting information

S1 File Study questionnaire. (DOCX)

S2 File Study questionnaire in English. (DOCX)
