# Supplementary material for: Penile implants and other high risk practices in French Guiana’s correctional facility: A cause for concern
Source: PLoS One. 2019 Jun 28;14(6):e0218992. doi: 10.1371/journal.pone.0218992 (PMC6599133; doi:10.1371/journal.pone.0218992)
Supplement: S1 File — (DOCX) [file pone.0218992.s001.docx]

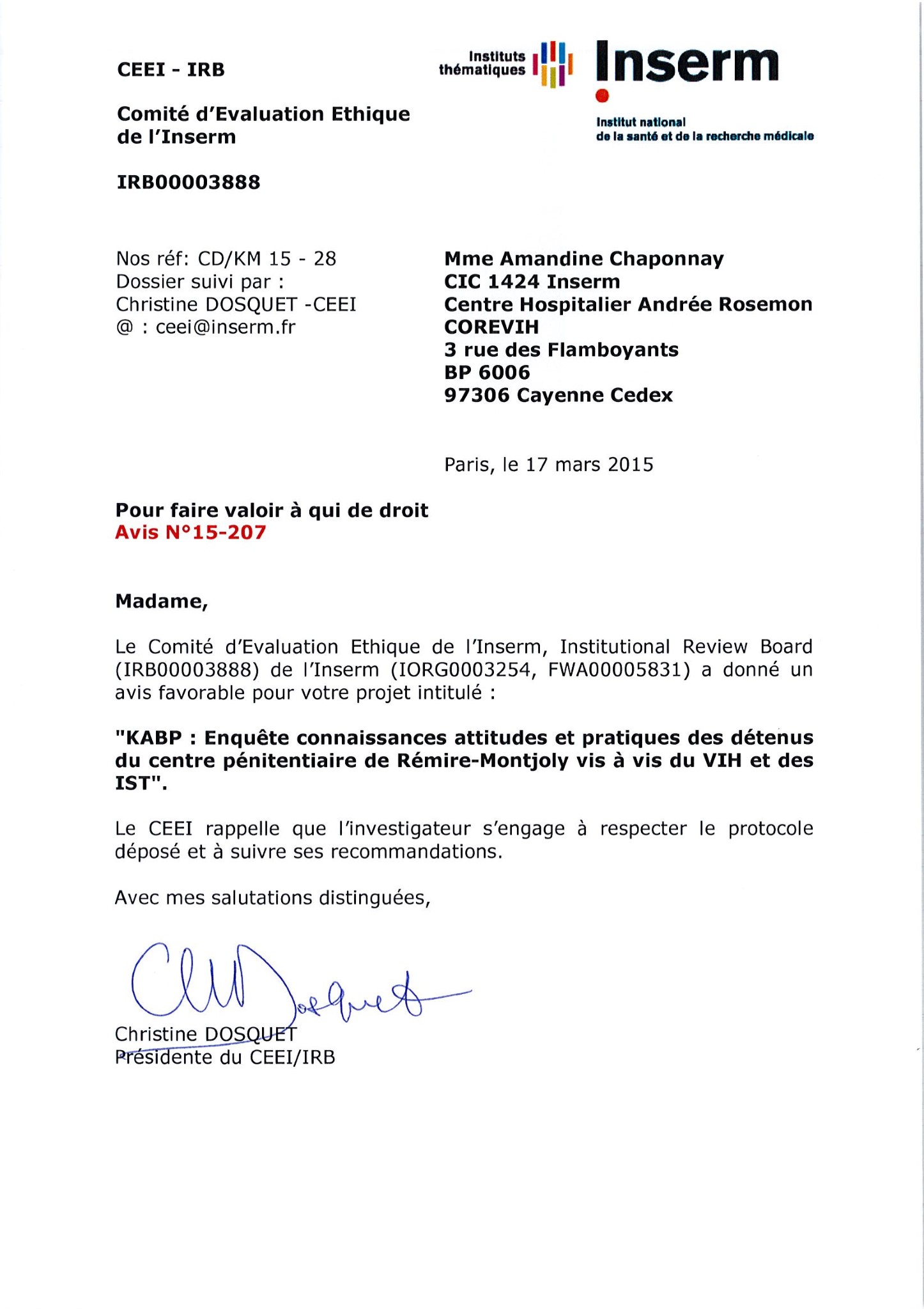


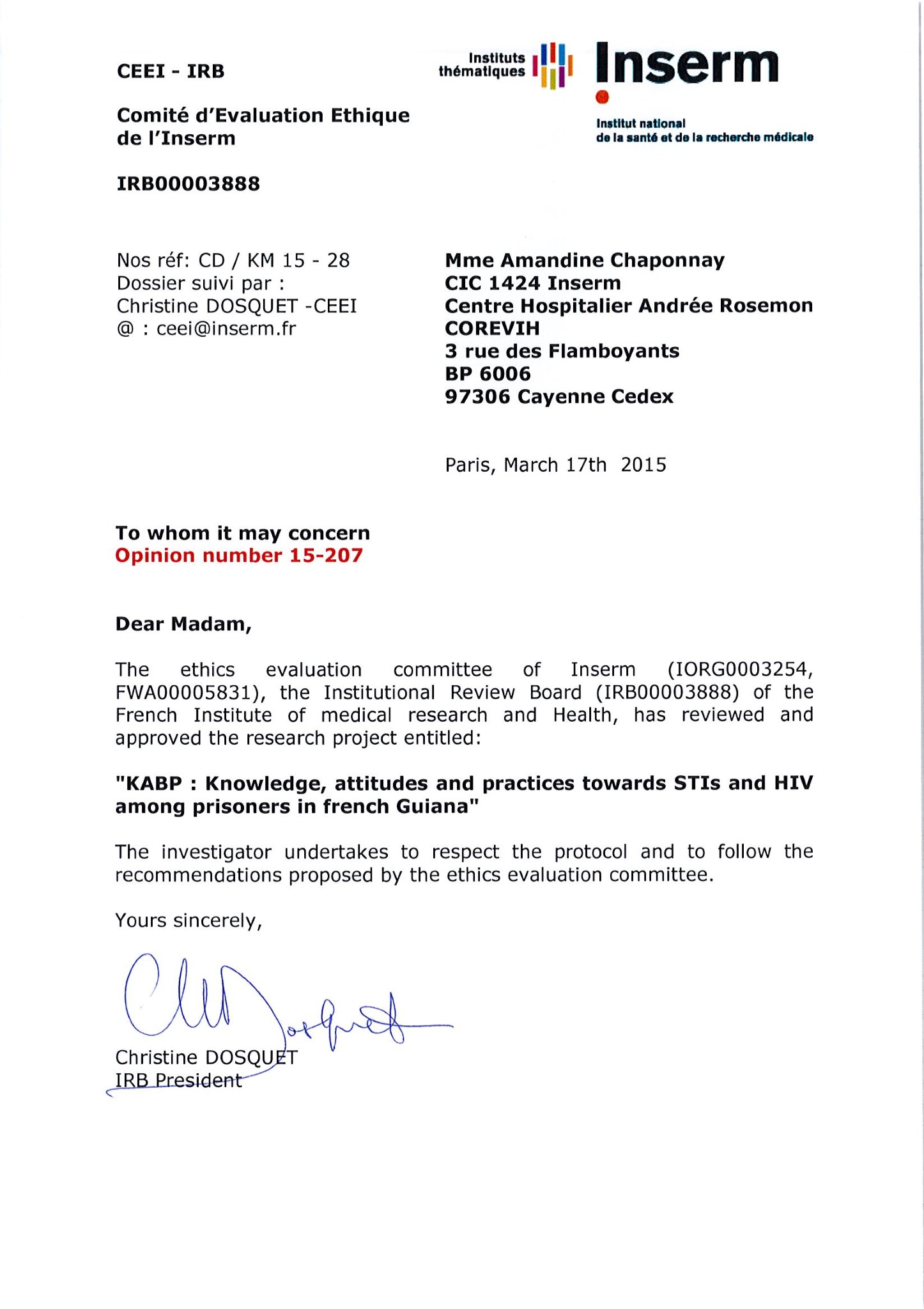


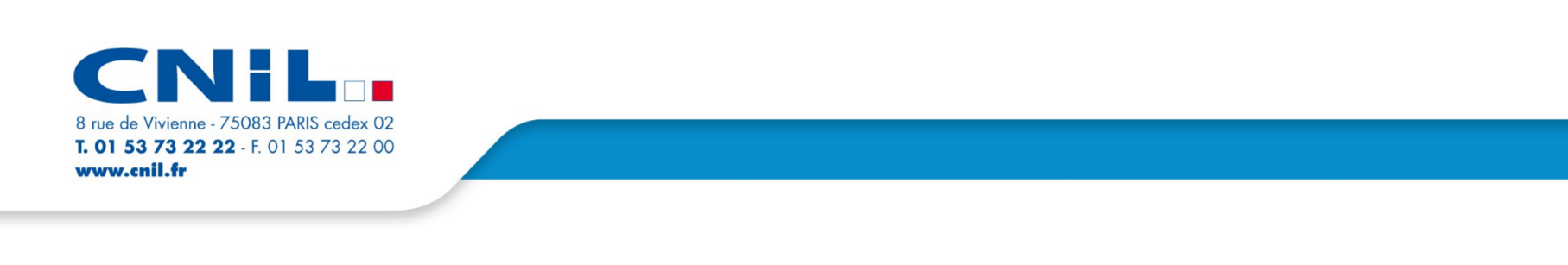
 Madame ROGIER Stéphanie

^RÉCÉPISSÉ^CENTRE HOSPITALIER ANDREE ROSEMON

INSERM 1424


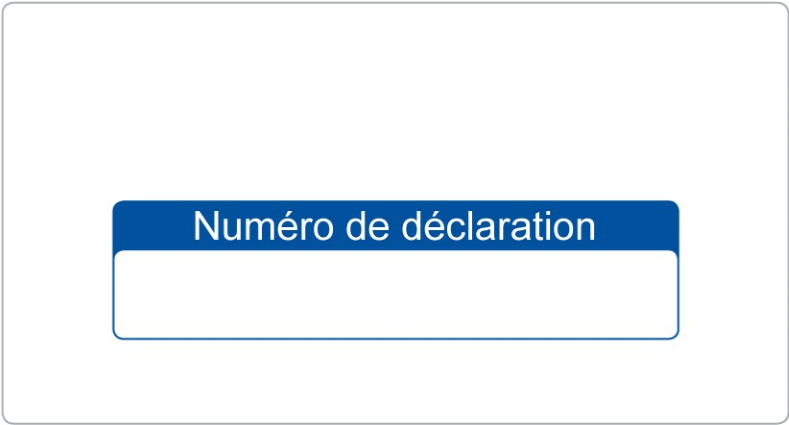


**1840401**

**v 0**

**DÉCLARATION NORMALE**

du 08 avril 2015

A LIRE IMPERATIVEMENT

3 RUE DES FLAMBOYANTS, BP 6006,

97306CAYENNE CEDEX

La délivrance de ce récépissé atteste que vous avez effectué une déclaration de votre traitement à la CNIL et que votre dossier est formellement complet. Vous pouvez mettre en œuvre votre traitement. Cependant, la CNIL peut à tout moment vérifier, par courrier ou par la voie d’un contrôle sur place, que ce traitement respecte l’ensemble des dispositions de la loi du 6 janvier 1978 modifiée en 2004. En tout état de cause, vous êtes tenu de respecter les obligations prévues par la loi et notamment :

1. La définition et le respect de la finalité du traitement,
2. La pertinence des données traitées,
3. La conservation pendant une durée limitée des données,
4. La sécurité et la confidentialité des données,
5. Le respect des droits des intéressés : information sur leur droit d’accès, de rectification et d’opposition.


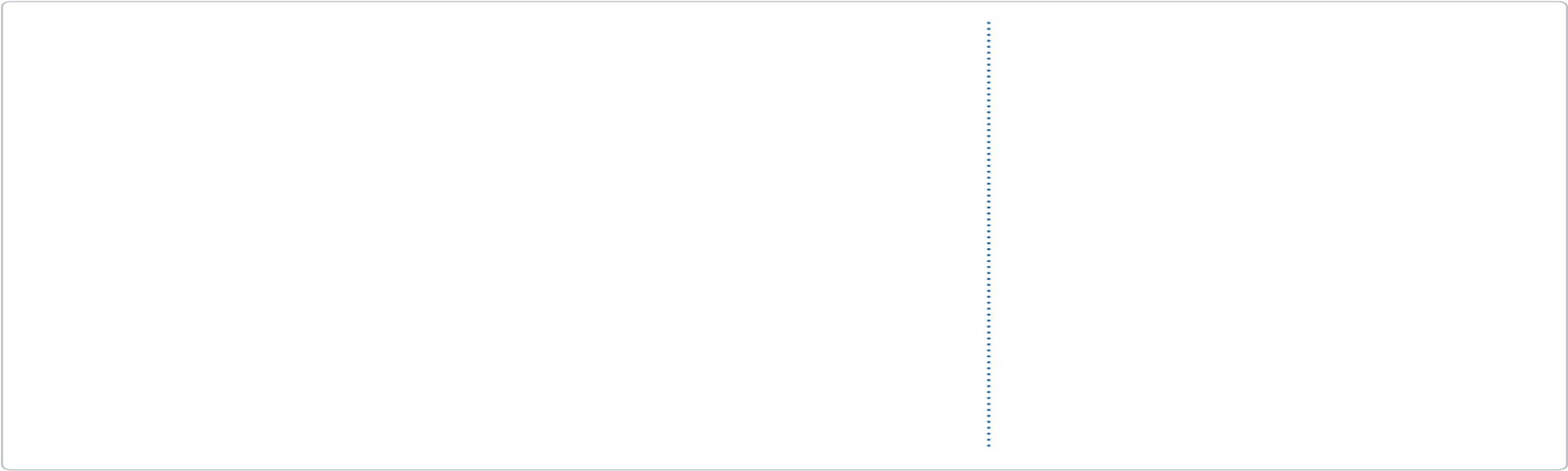


Fait à Paris,

le

2015

avril

08

**Nom**

:

CENTRE HOSPITALIER ANDREE ROSEMON

**Service**

:

CENTRE D'INVESTIGATION CLINIQUE

**Adresse**

:

RUE DES FLAMBOYANTS, BP 6006,

3

**Code postal**

:

97306

**Ville**

:

CAYENNE CEDEX


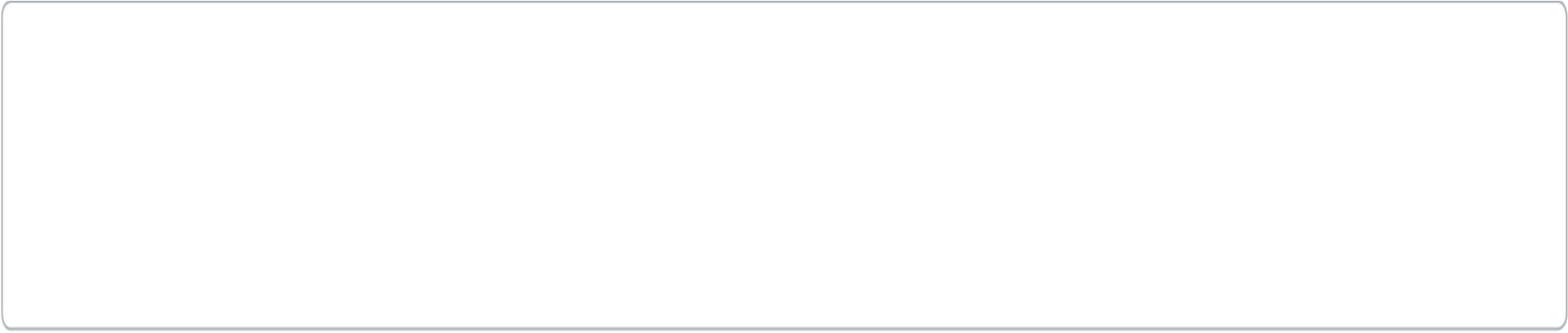


**Finalité**

**:**

DECRIRE LES CONNAISSANCES, ATTITUDES ET PRATIQUES DES DETENUS DU CENTRE

PENITENTIAIRE DE REMIRE-MONTJOLYVIS-A-VIS DU VIH ET LES IST.


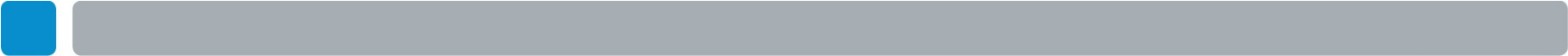

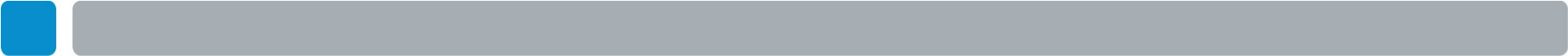


**Traitement déclaré**

**Organisme déclarant**

**N° SIREN ou SIRET :**

269733028

00022

**Code NAF ou APE :**

Z

8610

**Tél.**

:

0594 39 50

50

**Fax.**

:

72

94 39 48

05

Par délégation de la commission


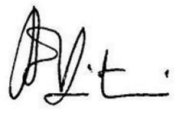


Isabelle FALQUE PIERROTIN Présidente


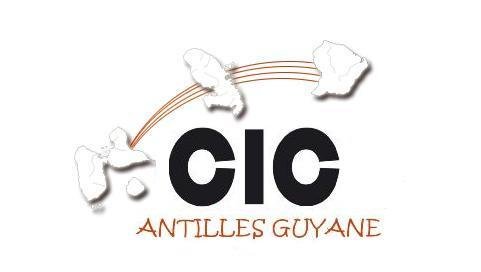
Annexe XX: Questionnaire


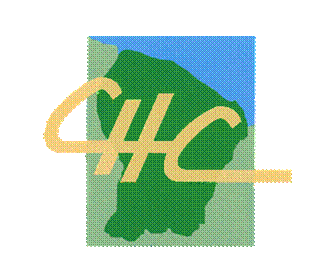
**
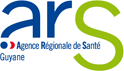
**
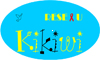

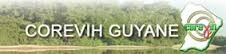


**Connaissances Attitudes Pratiques**

**des détenus face au VIH/SIDA et IST**

**N° de questionnaire (**Numéro aléatoire séquentiel) **:**

| \|__\|\|__\|\|____\|__\|__\|\|__\| |
| --- |

**Date de l'entretien :**

|__||__||__||__||__||__|

**Introduction des enquêteurs**:

Bonjour je m'appelle.....je suis ….je travaille dans le cadre d'un projet « VIH-prison », en association avec l’association Kikiwi, nous étudions les connaissances des détenus sur le VIH et les infections sexuellement transmissibles dans le but d'améliorer les messages de prévention en milieu carcéral.

Si vous êtes d'accord pour répondre à mes questions, ce questionnaire dure une cinquantaine de minutes, il est anonyme, vous êtes libre de refuser.

Ce questionnaire est proposé au hasard à un grand nombre de détenus, afin de mieux comprendre votre milieu et identifier les problèmes liés à la santé, et permettre à terme de mieux vous aider.

Je vous remercie de répondre avec sincérité à ce questionnaire.

Signature de l'enquêteur :

**1-CARACTERISTIQUES GENERALES**

**« Je vais vous poser des questions générales sur vous »**

| **N°** | **Questions** | **Catégories de codification** | **Aller à** |
| --- | --- | --- | --- |
| 1 | Sexe de la personne interrogée | Homme : 1  Femme : 2  Transgenre : 3 |  |
| 2 | Quel âge avez-vous ? | Age en années révolues \|__\|\|__\|  Ne sait pas : 88  Pas de réponse : 99 |  |
| 3 | Quel est votre pays de naissance ? | France : 1  Suriname : 2  Haïti : 3  Brésil : 4  Guyana : 5  République dominicaine : 6  Sainte-Lucie : 7  Autre : 8  Si autre précisez :…………………  Pas de réponse : 99 |  |
| 4 | Depuis combien d’années vivez-vous en Guyane ? | Nombre d’années révolues \|__\| :1  Depuis toujours : 2  Ne sais pas : 88  Pas de réponse : 99 |  |
| 5 | Quelle est votre langue maternelle ? | Français : 1  Hollandais : 2  Bushi nengue tongoe : 3  Brésilien (portugais) : 4  Anglais : 5  Espagnol : 6  Créole Haïtien : 7  Créole Guyanais : 8  Autre : précisez……………… : 9  Pas de réponse : 99 |  |
| 6 | Savez-vous lire/écrire ? | Oui, les deux : 1  Oui, juste lire : 2  Oui, juste écrire : 3  Non : 4  Pas de réponse : 99 |  |
| 7 | Jusqu’à quel âge êtes-vous allé à l’école ? | Age en années \|__\|\|__\|  Etudes en cours : 1  Ne sait pas : 88  Pas de réponse : 99 |  |
| 8 | Quel est le diplôme le plus élevé que vous ayez obtenu ? | CAP/BEP/BEPC : 1  Baccalauréat : 2  Etudes supérieures : 3  Sans diplôme, arrêt avant 16 ans : 4  Sans diplôme, arrêt après 16 ans : 5  Autre : précisez……………….. : 6  Jamais scolarisé : 7  Ne sait pas : 88  Pas de réponse : 99 |  |
| 9 | Où logiez vous le mois précédant votre incarcération ? | Maison familiale : 1  Maison personnelle : 2  Maison, appartement loué : 3  Collocation : 4  Logement social : 5  Chambre chez un particulier : 6  Habitat spontané (cabanes..) : 7  Squat : 8  Sans domicile fixe : 9  Dans la rue : 10  Autre : précisez…………… : 11  Pas de réponse : 99 |  |
| 10 | Dans quelle commune et quel quartier habitiez-vous le mois précédent votre incarcération ? | Commune :  Quartier : |  |
| 11 | Quelle était votre situation juste avant l’incarcération ? | Marié : 1  En couple, habitant ensemble : 2  En couple, n’habitant pas ensemble: 3  Célibataire : 4  Veuf : 5  Divorcé : 6  Pas de réponse : 99 |  |
| 12 | Avez-vous des enfants ? | Oui : 1  Non : 2  Non réponse : 99 |  |
| 13 | Avant votre incarcération, quelles ont été vos ressources personnelles ?  (réponses multiples possibles) | Ressources liées à une activité professionnelle déclarée, précisez  laquelle : …………………… : 1  Ressources liée à des jobs, produits de la pêche, de l'abattis : 2  Soutien familial : 3  Allocation adulte handicapé : 4  Allocations chômage : 5  RSA : 6  Revenu de retraite, minimum vieillesse : 7  Aucun : 8  De la manche : 9  Travail du sexe : 10  Vol : 11  Autre : précisez…………… : 12  Pas de réponse : 99 |  |
| 14 | Est-ce que la religion dans votre vie est : | Très importante : 1  Importante : 2  Pas très importante : 3  Pas importante du tout : 4  Ne sait pas : 88  Pas de réponse : 99 |  |

**2-VIE CARCERALE**

**« Je vais maintenant vous poser quelques questions sur vos conditions de vie en détention »**

| 15 | Dans quelle unité êtes-vous ? | Centre de détention : 1  Maison d'arrêt : 2  Quartier femmes : 3 |  |
| --- | --- | --- | --- |
| 16 | S'agit-il de votre première incarcération ? | Oui : 1  Non : 2  Pas de réponse | **Q18** |
| 17 | Si NON, combien de fois avez-vous déjà été incarcéré ? | 1 fois : 1  2 fois : 2  3 fois : 3  fois : 4  >5fois : 5  Ne sait pas : 88  Pas de réponse : 99 |  |
| 18 | Pour cette incarcération, depuis combien de temps êtes-vous incarcéré ? | Nombre de mois : _______  Ne sait pas : 88  Pas de réponse : 99 |  |
| 19 | Dans votre vie, combien de temps avez-vous passé au total en prison ? | Nombre de mois : _______  Ne sait pas : 88  Pas de réponse : 99 |  |
| 20 | Avec combien de personnes partagez-vous votre cellule ? | 0 : 1  1 : 2  2 : 3  3 : 4  4 : 5  5 : 6  Pas de réponse : 99 |  |

**3-ETAT DES LIEUX DES CONSOMMATIONS et COMORBIDITES PSYCHIATRIQUES**

**« Je vais vous poser quelques questions sur vos consommations d'alcool, de drogues et vos antécédents psychiatriques »**

| 21 | **Avant incarcération**, combien de fois vous arrivait-t-il de consommer de l’alcool ? | Jamais : 1  Une fois par mois : 2  2 à 4 fois par mois : 3  2 à 3 fois par semaine : 4  4 fois ou plus par semaine : 5  Ne sait pas : 88  Pas de réponse : 99 |  |  |
| --- | --- | --- | --- | --- |
| 22 | **Avant incarcération,** combien de verres (ou canettes) standard buviez-vous au cours d’une journée ordinaire où vous buviez de l’alcool ? | Un ou deux : 1  Trois ou quatre : 2  Cinq ou six : 3  Sept à neuf : 4  Dix ou plus : 5 |  |  |
| 23 | **Avant incarcération, c**ombien de fois vous arrivait-t-il de boire 6 verres standard ou davantage au cours d’une même occasion ? | Jamais : 1  Moins d’une fois par mois : 2  Une fois par mois : 3  Une fois par semaine : 4  Chaque jour ou presque : 5  Ne sait pas : 88  Pas de réponse : 99 |  |  |
| 24 | **Avant incarcération,** quelle boisson buviez-vous le plus souvent ? | Rhum : 1  Bière : 2  Whisky : 3  Bita : 4  Autre : précisez : _____________ : 5  Ne sait pas : 88  Pas de réponse : 99 |  |  |
| 25 | **Depuis l’incarcération**, combien de fois vous arrive-t-il de consommer de l’alcool ? | Jamais : 1  Une fois par mois : 2  2 à 4 fois par mois : 3  2 à 3 fois par semaine : 4  4 fois ou plus par semaine : 5  Ne sait pas : 88  Pas de réponse : 99 |  |  |
| 26 | **Depuis l’incarcération,** combien de verres (ou canettes) standard buvez-vous au cours d’une journée ordinaire où vous buvez de l’alcool ? | Un ou deux : 1  Trois ou quatre : 2  Cinq ou six : 3  Sept à neuf : 4  Dix ou plus : 5 |  |  |
| 27 | **Depuis l’incarcération, c**ombien de fois vous arrive-t-il de boire 6 verres standard ou davantage au cours d’une même occasion ? | Jamais : 1  Moins d’une fois par mois : 2  Une fois par mois : 3  Une fois par semaine : 4  Chaque jour ou presque : 5  Ne sait pas : 88  Pas de réponse : 99 |  |  |
| 28 | **Depuis l’incarcération,**quelle boisson vous arrive-t-il de boire le plus souvent ? | Rhum : 1  Bière : 2  Whisky : 3  Bita : 4  Autre : précisez : _____________ : 5  Ne sait pas : 88  Pas de réponse : 99 |  |  |
| 29 | **Si vous consommez des drogues,** avant l'incarcération, avez vous consommé…  Kali | Tous les jours : 1  Au moins une fois par semaine : 2  Moins d’une fois par semaine : 3  Jamais : 4  Ne sait pas : 5  Pas de réponse: 6 |  |  |
| 30 | Crack | Tous les jours : 1  Au moins une fois par semaine : 2  Moins d’une fois par semaine : 3  Jamais : 4  Ne sait pas : 5  Pas de réponse : 6 |  |  |
| 31 | Cocaïne | Tous les jours : 1  Au moins une fois par semaine : 2  Moins d’une fois par semaine : 3  Jamais : 4  Ne sait pas : 88  Pas de réponse : 99 |  |  |
| 32 | Blaka : (black joint : cannabis+crack) | Tous les jours : 1  Au moins une fois par semaine : 2  Moins d’une fois par semaine : 3  Jamais : 4  Ne sait pas : 88  Pas de réponse : 99 |  |  |
| 33 | Autres :  Précisez : ……………….. | Tous les jours : 1  Au moins une fois par semaine : 2  Moins d’une fois par semaine : 3  Jamais : 4  Ne sait pas : 88  Pas de réponse : 99 |  |  |
| 34 | **Si vous consommez des drogues,** depuis l'incarcération, avez vous consommé…  Kali | Tous les jours : 1  Au moins une fois par semaine : 2  Moins d’une fois par semaine : 3  Jamais : 4  Ne sait pas : 88  Pas de réponse : 99 |  | |
| 35 | Crack | Tous les jours : 1  Au moins une fois par semaine : 2  Moins d’une fois par semaine : 3  Jamais : 4  Ne sait pas : 88  Pas de réponse : 99 |  | |
| 36 | Cocaïne | Tous les jours : 1  Au moins une fois par semaine : 2  Moins d’une fois par semaine : 3  Jamais : 4  Ne sait pas : 88  Pas de réponse : 99 |  | |
| 37 | Blaka : (black joint : cannabis+crack) | Tous les jours : 1  Au moins une fois par semaine : 2  Moins d’une fois par semaine : 3  Jamais : 4  Ne sait pas : 88  Pas de réponse : 99 |  | |
| 38 | Autres : Précisez…………... | Tous les jours : 1  Au moins une fois par semaine : 2  Moins d’une fois par semaine : 3  Jamais : 4  Ne sait pas : 88  Pas de réponse : 99 |  | |
| 39 | Pensez-vous qu’il vous serait difficile ou facile d’obtenir du cannabis dans les 24 heures si vous en vouliez ? | Impossible à obtenir : 1  Très difficile à obtenir : 2  Assez difficile à obtenir : 3  Assez facile à obtenir : 4  Très facile à obtenir : 5  Ne sait pas : 88  Pas de réponse : 99 |  | |
| 40 | Dans votre vie avez- vous déjà été hospitalisé pour des problèmes psychiatriques ? | Oui : 1  Non : 2  Ne sait pas : 88  Pas de réponse : 99 |  | |
| 41 | Avez-vous déjà été suivi ou pris un traitement à l'UFPI ? (service psychiatrique de la prison) | Oui : 1  Non : 2  Ne sait pas : 88  Pas de réponse : 99 |  | |

**4-CONNAISSANCES, ATTITUDES et OPINIONS SUR LE VIH**

| 42 | Parmi ces différents risques et maladies, pouvez-vous me dire si vous les craignez pour vous-même, pas du tout, peu, pas mal, beaucoup ?  Le cancer | Pas du tout : 1  Peu : 2  Pas mal : 3  Beaucoup : 4  Ne sait pas : 88 |  |
| --- | --- | --- | --- |
| 43 | Le VIH/sida | Pas du tout : 1  Peu : 2  Pas mal : 3  Beaucoup : 4  Ne sait pas : 88 |  |
| 44 | La dengue | Pas du tout : 1  Peu : 2  Pas mal : 3  Beaucoup : 4  Ne sait pas : 88 |  |
| 45 | Les risques liés aux pesticides | Pas du tout : 1  Peu : 2  Pas mal : 3  Beaucoup : 4  Ne sait pas : 88 |  |
| 46 | La stérilité (le fait de ne pas pouvoir avoir d’enfants) | Pas du tout : 1  Peu : 2  Pas mal : 3  Beaucoup : 4  Ne sait pas : 88 |  |
| 47 | Poser si femme : Tomber enceinte sans l’avoir prévu | Pas du tout : 1  Peu : 2  Pas mal : 3  Beaucoup : 4  Ne sait pas : 88 |  |
| 48 | Poser si hommes : Qu’une de vos partenaires tombe enceinte sans que vous l’ayez prévu | Pas du tout : 1  Peu : 2  Pas mal : 3  Beaucoup : 4  Ne sait pas : 88 |  |
| ***A. CONNAISSANCES*** | | | |
| 49 | Pouvez-vous indiquer si oui ou non le virus du sida peut être transmis dans chacune des circonstances suivantes :  Lors de rapports sexuels sans préservatif | Oui : 1  Non : 2  Ne sait pas : 88  Pas de réponse : 99 |  |
| 50 | Lors de rapports sexuels avec préservatifs | Oui : 1  Non : 2  Ne sait pas : 88  Pas de réponse : 99 |  |
| 51 | Dans les toilettes publiques | Oui : 1  Non : 2  Ne sait pas : 88  Pas de réponse : 99 |  |
| 52 | En buvant dans le verre d'une personne contaminée | Oui : 1  Non : 2  Ne sait pas : 88  Pas de réponse : 99 |  |
| 53 | Par une piqûre de moustique | Oui : 1  Non : 2  Ne sait pas : 88  Pas de réponse : 99 |  |
| 54 | Lors d'une piqûre de drogue avec une seringue déjà utilisée | Oui : 1  Non : 2  Ne sait pas : 88  Pas de réponse : 99 |  |
| 55 | En embrassant une personne contaminée | Oui : 1  Non : 2  Ne sait pas : 88  Pas de réponse : 99 |  |
| 56 | De la mère à son enfant au moment de la grossesse ou de l’allaitement | Oui : 1  Non : 2  Ne sait pas : 88  Pas de réponse : 99 |  |
| 57 | Je vais vous citer un certain nombre de moyens éventuels pour se protéger du sida. Pour chacun d'entre eux, pensez-vous un moyen tout à fait efficace, plutôt, plutôt pas ou pas du tout efficace  Se laver après l'acte sexuel | Tout à fait efficace : 1  Plutôt efficace : 2  Peu efficace : 3  Pas du tout efficace : 4  Ne sait pas : 88  Pas de réponse : 99 |  |
| 58 | Avoir des rapports sexuels avec peu de partenaires différents | Tout à fait efficace : 1  Plutôt efficace : 2  Peu efficace : 3  Pas du tout efficace : 4  Ne sait pas : 88  Pas de réponse : 99 |  |
| 59 | Utiliser un préservatif masculin | Tout à fait efficace : 1  Plutôt efficace : 2  Peu efficace : 3  Pas du tout efficace : 4  Ne sait pas : 88  Pas de réponse : 99 |  |
| 60 | Demander un test de dépistage du virus du Sida à ses partenaires | Tout à fait efficace : 1  Plutôt efficace : 2  Peu efficace : 3  Pas du tout efficace : 4  Ne sait pas : 88  Pas de réponse : 99 |  |
| 61 | Faire régulièrement un test de dépistage du virus du sida | Tout à fait efficace : 1  Plutôt efficace : 2  Peu efficace : 3  Pas du tout efficace : 4  Ne sait pas : 88  Pas de réponse : 99 |  |
| 62 | Se retirer avant la fin du rapport sexuel | Tout à fait efficace : 1  Plutôt efficace : 2  Peu efficace : 3  Pas du tout efficace : 4  Ne sait pas : 88  Pas de réponse : 99 |  |
| 63 | Choisir des partenaires que l’on pense sans risque | Tout à fait efficace : 1  Plutôt efficace : 2  Peu efficace : 3  Pas du tout efficace : 4  Ne sait pas : 88  Pas de réponse : 99 |  |
| 64 | La pénétration anale transmet moins le VIH que la pénétration vaginale ? | Vrai : 1  Faux : 2  Ne sait pas : 88  Pas de réponse : 99 |  |

***B. ATTITUDES, STIGMATISATIONS VIS A VIS DES PERSONNES SEROPOSITIVES***

**« Nous allons parler de ce que pense la société, la communauté, la famille, des personnes atteintes du VIH »**

Si vous saviez qu’une personne était contaminée par le VIH, accepteriez-vous :

| 65 | De travailler avec elle ? |  | Oui : 1  Non : 2  Peut être : 77  Ne sait pas : 88  Pas de réponse : 99 |  |
| --- | --- | --- | --- | --- |
| 66 | De manger avec elle ? |  | Oui : 1  Non : 2  Peut être : 77  Ne sait pas : 88  Pas de réponse : 99 |  |
| 67 | Qu'elle fasseà la cuisine ? |  | Oui : 1  Non : 2  Peut être : 77  Ne sait pas : 88  Pas de réponse : 99 |  |
| 68 | De partager votre cellule avec elle ? |  | Oui : 1  Non : 2  Peut être : 77  Ne sait pas : 88  Pas de réponse : 99 |  |
| 69 | D'avoir des relations sexuelles avec elle en utilisant des préservatifs ? |  | Oui : 1  Non : 2  Peut être : 77  Ne sait pas : 88  Pas de réponse : 99 |  |
| **C.OPINIONS SUR LE VIH ET LEPRESERVATIF** | | | | |
| 70 | Vous sentez-vous personnellement concerné(e) par les campagnes d'information sur le sida et les préservatifs ? |  | Pas du tout concerné(e) : 1  Un peu concerné(e) : 2  Très concerné(e) : 3  Ne sait pas : 88  Pas de réponse : 99 |  |
| 71 | Je vais vous demander votre avis sur un certain nombre de thèmes de débat qui traversent la société. Pour chacun d'eux, pouvez-vous me dire si vous êtes tout à fait d’accord, plutôt, plutôt pas ou pas du tout d’accord ?  Il faut interdire à un enfant atteint du virus du sida d'aller à l'école : |  | Tout à fait d'accord : 1  Plutôt d'accord : 2  Plutôt pas d'accord : 3  Pas du tout d'accord : 4  Ne sait pas : 88  Pas de réponse : 99 |  |
| 72 | Les homosexuels sont des gens comme les autres : |  | Tout à fait d'accord : 1  Plutôt d'accord : 2  Plutôt pas d'accord : 3  Pas du tout d'accord : 4  Ne sait pas : 88  Pas de réponse : 99 |  |
| 73 | Il faut isoler les malades du sida du reste de la population : |  | Tout à fait d'accord : 1  Plutôt d'accord : 2  Plutôt pas d'accord : 3  Pas du tout d'accord : 4  Ne sait pas : 88  Pas de réponse : 99 |  |
| 74 | Il faut développer les programmes d'éducation sexuelle dans les écoles : |  | Tout à fait d'accord : 1  Plutôt d'accord : 2  Plutôt pas d'accord : 3  Pas du tout d'accord : 4  Ne sait pas : 88  Pas de réponse : 99 |  |
| 75 | Le sida est un châtiment de Dieu, une malédiction, un mauvais sort : |  | Tout à fait d'accord : 1  Plutôt d'accord : 2  Plutôt pas d'accord : 3  Pas du tout d'accord : 4  Ne sait pas : 88  Pas de réponse : 99 |  |

**« Je vais vous poser quelques questions sur les préservatifs masculins »**

|  | Pour chacune des opinions suivantes sur le préservatif, pouvez-vous me dire si vous êtes d'accord ou pas d'accord ? | |  |
| --- | --- | --- | --- |
| 76 | Le préservatif, c'est pour les jeunes | Tout à fait d'accord : 1  Plutôt d'accord : 2  Plutôt pas d'accord : 3  Pas du tout d'accord : 4  Ne sait pas : 88  Pas de réponse : 99 |  |
| 77 | Le préservatif ça permet d’avoir des rapports sans se poser de question | Tout à fait d'accord : 1  Plutôt d'accord : 2  Plutôt pas d'accord : 3  Pas du tout d'accord : 4  Ne sait pas : 88  Pas de réponse : 99 |  |
| 78 | Le préservatif, c'est compliqué à utiliser avec un même partenaire sur une longue période | Tout à fait d'accord : 1  Plutôt d'accord : 2  Plutôt pas d'accord : 3  Pas du tout d'accord : 4  Ne sait pas : 88  Pas de réponse : 99 |  |
| 79 | Le préservatif, ça crée des doutes sur le partenaire | Tout à fait d'accord : 1  Plutôt d'accord : 2  Plutôt pas d'accord : 3  Pas du tout d'accord : 4  Ne sait pas : 88  Pas de réponse : 99 |  |
| 80 | Utiliser un préservatif, c'est quelque chose de banal | Tout à fait d'accord : 1  Plutôt d'accord : 2  Plutôt pas d'accord : 3  Pas du tout d'accord : 4  Ne sait pas : 88  Pas de réponse : 99 |  |
| 81 | Le préservatif, c'est difficile d’en utiliser systématiquement | Tout à fait d'accord : 1  Plutôt d'accord : 2  Plutôt pas d'accord : 3  Pas du tout d'accord : 4  Ne sait pas : 88  Pas de réponse : 99 |  |
| 82 | Est-il facile de se procurer des préservatifs à la prison ? | Oui : 1  Non : 2  Ne sait pas : 88  Pas de réponse : 99 |  |
| 83 | Si NON, pourquoi ? | Il n'y en a pas : 1  J'ai honte d'en prendre : 2  Je ne sais pas où ils sont : 3  Ils ne sont pas placés au bon endroit : 4  Autre, précisez………………… : 5  Ne sait pas : 88  Pas de réponse : 99 |  |

***D. TRAITEMENTS DU VIH et DU SIDA***

**« Nous allons parler des traitements disponibles »**

| 84 | Avez-vous déjà entendu parler des traitements pour le VIH/SIDA | Oui : 1  Non : 2  Ne sait pas : 88  Pas de réponse : 99 |  |
| --- | --- | --- | --- |
| 85 | Grâce à ces traitements, les personnes séropositives pour le VIH peuvent vivre normalement | Oui : 1  Non : 2  Ne sait pas : 88  Pas de réponse : 99 |  |
| 86 | Grâce à ces traitements, on guérit définitivement du SIDA | Oui : 1  Non : 2  Ne sait pas : 88  Pas de réponse : 99 |  |
| 87 | Les personnes infectées par le VIH qui prennent un traitement adapté transmettent moins le VIH | Oui : 1  Non : 2  Ne sait pas : 88  Pas de réponse : 99 |  |
| 88 | Avez-vous entendu parler du traitement post exposition (un traitement d'urgence)qui, pris juste après un rapport sexuel non protégé, peut réduire le risque d’être contaminé par le virus du sida ? | Oui : 1  Non : 2  Ne sait pas : 88  Pas de réponse : 99 |  |
| 89 | Avez-vous déjà entendu parler de la circoncision ? (ablation du prépuce) | Oui : 1  Non : 2  Ne sait pas : 88  Pas de réponse : 99 |  |
| 90 | La circoncision diminue de 60% le risque de contamination pour le VIH, cela vous semblerait-il une solution acceptable si on vous la proposait ? | Oui : 1  Non : 2  Ne sait pas : 88  Pas de réponse : 99 |  |

***E. PERCEPTION DU RISQUE d'être infecté par le VIH***

« Nous allons maintenant parler du risque d'être infecté par le VIH »

| 91 | Vous-même, vous considérez que par rapport à la moyenne des gens, vous avez plus de risque, le même risque, moins de risque ou aucun risque d'être contaminé par le virus du sida ? | Plus de risque : 1  Le même risque : 2  Moins de risque : 3  Aucun risque : 4  Ne sait pas : 88  Pas de réponse : 99 |  |
| --- | --- | --- | --- |
| 92 | Vous-même, avez-vous déjà craint d’avoir été contaminé par le virus du sida ? | Oui, plusieurs fois : 1  Oui, une fois : 2  Non : 3  Ne sait pas : 88  Pas de réponse : 99 |  |

**5-HISTOIRE SEXUELLE**

***A. PREMIER RAPPORT SEXUEL***

**« Nous allons parler du premier rapport sexuel que vous avez eu dans votre vie »**

| 93 | A quel âge avez vous eu votre premier rapport sexuel? |  | Age en années [__\|__]  Ne sait pas : 88  Pas de réponse : 99  N’en a jamais eu : 77 |  |
| --- | --- | --- | --- | --- |
| 94 | Ce premier rapport sexuel était quelque chose : |  | Que vous souhaitiez à ce moment là : 1  Que vous avez accepté mais que vous ne souhaitiez pas  vraiment : 2  Que vous avez été forcé à faire contre votre volonté : 3  Pas de réponse : 99 |  |
| 95 | Lors de ce premier rapport sexuel, avez vous utilisé un préservatif ? |  | Oui : 1  Non : 2  Ne sait pas : 88  Pas de réponse : 99 |  |
| 96 | Combien avez-vous eu de partenaires différents dans votre vie ? |  | 5 et moins : 1  10 et moins : 2  20 et moins : 3  Entre 20 et 40 : 4  Plus de 40 : 5  Ne sait pas: 88  Pas de réponse: 99 |  |
| 97 | Combien avez-vous eu de partenaires différents durant les 12 derniers mois ? |  | Total ____  Ne sait pas : 88  Pas de réponse : 99 |  |
| 98 | (à poser aux hommes)  Dans votre vie, vous avez eu des rapports sexuels… |  | Uniquement avec des femmes : 1  Avec des femmes et  des hommes : 2  Uniquement avec des  Hommes : 3  Non réponse : 99 |  |
| 99 | (à poser aux femmes)  Dans votre vie, vous avez eu des rapports sexuels… |  | Uniquement avec des hommes : 1  Avec des hommes et  des femmes : 2  Uniquement avec des femmes : 3  Pas de réponse : 99 |  |
| 100 | Au cours des cinq dernières années, avez-vous eu plusieurs partenaires durant la même période, c’est-à-dire commencer à avoir des rapports sexuels avec une personne tout en continuant à avoir des rapports sexuels avec une autre ? |  | Oui : 1  Non : 2  Pas de réponse : 99 |  |
| 101 | Et au cours des 12 derniers mois, avez-vous eu plusieurs partenaires durant la même période, c’est à dire commencer à avoir des rapports sexuels avec ne personne tout en continuant à avoir des rapports sexuels avec une autre ? |  | Oui : 1  Non : 2  Pas de réponse : 99 |  |
| 102 | Au cours des 5 dernières années, avez-vous eu, au moins une fois, des rapports sexuels en payant ou en étant payé ? |  | Oui, en payant : 1  Oui, en étant payé : 2  Non : 3  Pas de réponse : 99 |  |
| 103 | Au cours des 12 derniers mois, avez-vous eu des rapports sexuels en payant ou en étant payé ? |  | Oui, en payant : 1  Oui, en étant payé : 2  Non : 3  Pas de réponse : 99 |  |
| 104 | Lors de ce dernier rapport, avez-vous utilisé un préservatif ? |  | Oui : 1  Non : 2  Pas de réponse : 99 |  |

***B. VIE SEXUELLE CARCERALE***

**Nous allons vous poser quelques questions sur votre vie sexuelle dans la prison :**

| 105 | Avez-vous eu des relations sexuelles depuis votre incarcération ? | Oui : 1  Non : 2  Pas de réponse : 99 | Q106 |
| --- | --- | --- | --- |
| 106 | Votre dernier rapport sexuel était : | Avec une femme : 1  Avec un homme : 2  Pas de réponse : 99 |  |
| 107 | Où a eu lieu ce dernier rapport sexuel ?  (plusieurs réponses possibles) | Au parloir : 1  En prison (douches, cellule..) : 2  Autre, précisez………………… : 3  Pas de réponse : 99 |  |
| 108 | Avez-vous utilisé un préservatif lors de ce dernier rapport sexuel ? | Oui: 1  Non : 2  Pas de réponse : 99 |  |
| 109 | Avant détention, vous a-t-on déjà forcé à avoir un rapport sexuel ? | Oui : 1  Non : 2  Pas de réponse : 99 |  |
| 110 | En détention, vous a-t-on déjà forcé à avoir un rapport sexuel? | Oui : 1  Non : 2  Pas de réponse : 99 |  |

**6-IMPLANTS PENIENS et PRATIQUES SANGLANTES**

| 111 | Avez-vous déjà inséré ou implanté un objet sous la peau de votre pénis (dominos)? | Oui : 1  Non : 2  Pas de réponse : 99 | **Q112** |
| --- | --- | --- | --- |
| 112 | Si oui, combien ? (si 1 à la Q111) | Nombre : \|_ _\|_ _\| | Q113 |
| 113 | Avez-vous fait cela lorsque vous étiez en prison ? | Oui, tous : 1  Oui, pas tous : 2  Non, aucun : 3  Pas de réponse : 99 |  |
| 114 | La pose a-t-elle été pratiquée : | Seul : 1  A l'aide d'un tiers : 2  Pas de réponse : 99 |  |
| 115 | La pose a-t-elle été pratiquée de manière : | Gratuite : 1  Rémunérée : 2  Pas de réponse : 99 |  |
| 116 | Quel était le matériel utilisé pour la pose ? | Lame de rasoir : 1  Couvercle de boîte de conserve : 2  Autre, précisez………………. : 3  Pas de réponse : 99 |  |
| 117 | Le matériel était-il :  (deux réponses possibles) | Neuf : 1  Usagé : 2  Désinfecté : 3  Souillé (non désinfecté) : 4  Pas de réponse : 99 |  |
| 118 | Y-a-t-il eu des complications après la pose ? | Oui : 1  Non : 2  Ne sait pas : 88  Pas de réponse : 99 | **Q119** |
| 119 | Si oui, lesquelles ?  (si 1 à la Q 118) | Gonflement : 1  Douleur : 2  Fièvre : 3  Hémorragie : 4  Problèmes de bandaison : 5  Problèmes de cicatrisation : 6  Autre, précisez…………… : 7  Pad de réponse : 99 |  |
| 120 | Pourquoi mettez vous des bouglous ?  (ne pas citer les propositions) | Parce que je trouve ça beau : 1  Pour augmenter le plaisir sexuel  de mes partenaires : 2  Pour augmenter mon plaisir sexuel : 3  Pour rendre les rapports  sexuels douloureux : 4  Pour avoir une fille plus facilement : 5  Parce que je fais partie d’un clan : 6  Autre : précisez…………… : 7  Pas de réponse : 99 |  |
| 121 | Pensez-vous que les bouglous gênent l’utilisation du préservatif ? (difficile à mettre, davantage de rupture de préservatif) | Oui : 1  Non : 2  Ne sait pas : 88  Pas de réponse : 99 |  |
| 122 | Vous est-il arrivé de mettre deux préservatifs l’un sur l’autre lors d’un rapport sexuel ? | Oui : 1  Non : 2  Ne sait pas : 88  Pas de réponse : 99 |  |
| 123 | Vous êtes vous fait un tatouage ou un piercing pendant votre incarcération ? | Oui : 1  Non : 2  Ne sait pas : 88  Pas de réponse : 99 |  |

**7-INFECTION SEXUELLEMENT TRANSMISSIBLES (IST)**

**«  Nous allons parler maintenant des maladies qui peuvent se transmettre par les rapports sexuels»**

| 124 | Combien de fois dans votre vie avez-vous déjà eu une infection ou maladie qui se transmet sexuellement ? | Nombre de fois______ |  |
| --- | --- | --- | --- |
| 125 | Nous allons parler de la dernière fois, de quand date cette maladie ? | Moins d’un an : 1  Plus d’un an mais moins  de 5 ans : 2  5 ans et plus : 3  Pas de réponse : 99 |  |
| 126 | De quelle maladie ou infection s'agissait-il (dernière fois) ?  (consigne enquêteur : citer si nécessaire) | Mycose /champignons / candidose : 1  Chlamydiae : 2  Gonocoque /blennorragie /  chaude pisse : 3  Trichomonas : 4  Syphilis : 5  Papillomavirus /condylome / crêtes de coq : 6  Hépatite B : 7  Mycoplasme : 8  Herpès génital : 9  VIH / sida : 10  Autre : préciser ……………  : 11  Ne sait plus le nom : 12  Pas de réponse : 99 |  |
| 127 | Avez-vous prévenu votre ou vos partenaire(s) que vous aviez cette maladie ou infection ?  (consigne enquêteur : citer) | Oui : 1  Oui mais pas tous : 2  Non : 3  C’est lui ou l’un deux  qui m’a prévenu(e) : 4  Pas de réponse : 99 |  |

**8-DEPISTAGE DU VIH**

**« Nous allons parler du test de dépistage du VIH »**

| 128 | Au cours de votre vie, avez-vous déjà effectué un test de dépistage du virus du sida ?  (consigne enquêteur : si « Oui », relancer « Une fois ? Plusieurs fois ? ») | Oui, plusieurs fois : 1  Oui, une fois : 2  Non : 3  Ne sait pas : 88  Pas de réponse : 99 | Q132  Q132 |
| --- | --- | --- | --- |
| 129 | Avez-vous effectué un test au cours des 5 dernières années ?  (consigne enquêteur : si « Oui », relancer « Une fois ? Plusieurs fois ? ») | Oui, plusieurs fois : 1  Oui, une fois : 2  Non : 3  Ne sait pas : 88  Pas de réponse : 99 | Q132  Q132 |
| 130 | Et dans les 12 derniers mois ? (consigne enquêteur : si « Oui », relancer « Une fois ? Plusieurs fois ? » | Oui, plusieurs fois : 1  Oui, une fois : 2  Non : 3  Ne sait pas : 88  Pas de réponse : 99 | Q132  Q132 |
| 131 | Concernant votre statut sérologique, vous diriez que… | Vous êtes séronégatif  (vous n’êtes pas contaminé  par le virus du sida) : 1  Vous étiez séronégatif lors du dernier test mais vous n’êtes plus certain de l’être encore aujourd’hui : 2  Vous êtes séropositif (vous êtes contaminé par le virus du sida) : 3  Pas de réponse : 99 |  |
| 132 | Avez-vous réalisé un test de dépistage du virus du sida en prison ? | Oui : 1  Non : 2  Ne sait pas : 88  Pas de réponse : 99 | Q 134 |
| 133 | Vous a-t-on convoqué pour vous rendre le résultat de ce test ?  (si réponse 1 à la 132) | Oui : 1  Non : 2  Ne sait pas : 88  Pas de réponse : 99 |  |
| 134 | Connaissez-vous votre statut sérologique vis à vis de l’hépatite C ? | Oui : 1  Non : 2  Ne sait pas : 88  Pas de réponse : 99 | Q136 |
| 135 | Si oui, quel est-t-il ?  (si réponse 1 à la 134) | Négatif : 1  Positif : 2  Ne sait pas : 88  Pas de réponse : 99 |  |

**9-PROXIMITE A LA MALADIE ET AUX PERSONNES SEROPOSITIVES**

| 136 | Connaissez-vous, personnellement, dans votre entourage (famille, amis, collègues…) une ou plusieurs personnes séropositives ou malades du sida ? (consigne enquêteur : citer) | Oui, une personne : 1  Oui, plusieurs : 2  Non, aucune : 3  Ne sait pas : 88  Pas de réponse : 99 | Q 138  Q138 |
| --- | --- | --- | --- |
| 137 | S'agit-il…  (consigne enquêteur : si plusieurs personnes connues – relancer « Quelle autre personne ?»)    (si réponses 1 et 2 à la 137) | De votre partenaire sexuel actuel ou de l’un de vos partenaires actuel : 1  D’un parent : 2  D’un ami : 3  D’une personne avec qui vous avez eu des relations sexuelles dans le passé : 4  D’un(e) collègue de travail : 5  D’une connaissance : 6  De quelqu'un dont vous avez entendu parler sans le connaître personnellement) : 7  De vous-même : 8  Autre : préciser_______________ : 9  Pas de réponse : 99 |  |
| 138 | Avez-vous connaissance de personnes séropositives ou malades du sida dans la prison ? | Oui : 1  Non : 2  Pas de réponse : 99 | Q140 |
| 139 | Comment le savez-vous / le croyez-vous ?  (si réponse 1 à la 139) | Parce que cela se voit : 1  Parce que la personne m’a parlé de sa séropositivité : 2  Parce que quelqu’un me  l’a dit : 3  Autre : précisez : __________ : 88  Pas de réponse : 99 |  |

**10-INTERVENTIONS DE PREVENTION et UTILISATION DES MEDIAS**

**“ Nous allons parler des messages sur le VIH/SIDA que vous avez pu avoir par les médias. »**

| 140 | Avez-vous déjà lu, vu ou entendu des informations sur le VIH/SIDA ?  *(plusieurs réponses possibles)* | Dans les journaux : 1  A la télévision : 2  A la radio : 3  Affiches dans la rue : 4  Structures, associations : 5  Jamais : 6  Ne sait pas : 88  Pas de réponse : 99 |  |
| --- | --- | --- | --- |
| 141 | Le mois dernier, vous diriez plutôt que vous avez ….  -écouté la radio : | Tous les jours : 1  Au moins une fois par semaine : 2  Moins d’une fois par semaine : 3  Jamais : 4  Ne sait pas : 88  Pas de réponse : 99 |  |
| 142 | -regardé la TV : | Tous les jours : 1  Au moins une fois par semaine : 2  Moins d’une fois par semaine : 3  Jamais : 4  Ne sait pas : 88  Pas de réponse : 99 |  |
| 143 | Quelles stations de radio écoutez-vous ?  *(plusieurs réponses possibles)* | Guyane première : 1  Radio Gabriel : 2  NRJ : 3  KFM : 4  Mosaïk : 5  Trace FM : 6  Autre………………… : 7  Ne sait pas : 88  Pas de réponse : 99 |  |
| 144 | Quelles chaînes de télévision regardez-vous?  *(plusieurs réponses possibles)* | Guyane première : 1  Tempo : 2  France O : 3  Autre, précisez..................…. 4  Ne sait pas : 88  Pas de réponse : 99 |  |
| 145 | Sur quels thèmes aimeriez-vous avoir des informations ? | Sur les modes de transmission  du virus du sida : 1  Sur le test de dépistage  du virus du sida : 2  Sur les traitements face  au VIH/sida : 3  Sur les préservatifs : 4  Autre : précisez………………. :5  Ne sait pas : 88  Pas de réponse : 99 |  |
| 146 | Sur quels autres thèmes de santé ou quel autre sujet souhaiteriez-vous avoir des informations ? | Réponses libres :  Ne sait pas : 88  Pas de réponse : 99 |  |

ANNEXE : Questionnaire de refus


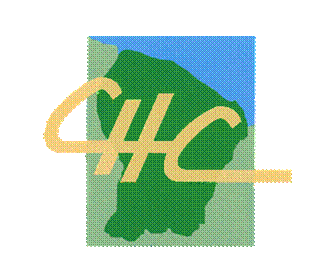
**
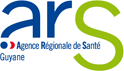
**
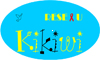

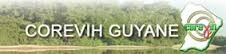

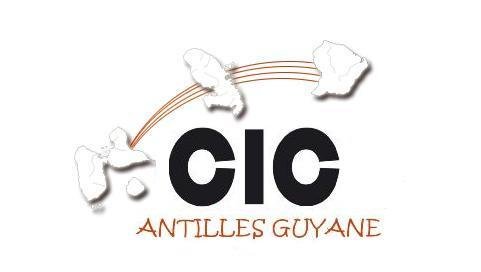


**QUESTIONNAIRE DE REFUS**

Titre de l’étude :

**Enquête connaissances attitudes et pratiquse des détenus du centre pénitentiaire de Rémire-Montjoly vis-à-vis du VIH et des IST**

Titre abrégé :

**KABP prison**

**Initiales enquêteurs** |__|__|__|__|**Fait le** _ _ /_ _/_ _ _ _

| **N°** | **Questions** | **Catégories de codification** |
| --- | --- | --- |
|  | Sexe de la personne interrogée | Homme 🞏  Femme 🞏  Transgenre 🞏  Pas de réponse 🞏 |
|  | Quel âge avez-vous ? | Âge en années révolues \|__\|__\|  Ne sait pas 🞏  Pas de réponse 🞏 |
|  | Quelle est votre nationalité ? | Française 🞏  Guyanienne 🞏  Surinamaise 🞏  Dominicaine 🞏  Brésilienne 🞏  Haïtienne 🞏  Dominiquaise 🞏  Sainte-Lucienne 🞏  Autre 🞏  Si autre, préciser : _______________________________  Pas de réponse 🞏 |
|  | Pourquoi ne souhaitez-vous pas répondre ?  ***Plusieurs réponses possibles*** | Je dois aller travailler 🞏  Je n’ai pas le temps 🞏  Je suis inquiet à propos du respect de mon anonymat 🞏  Je ne veux pas répondre 🞏  Ces questions sont trop personnelles, embarrassantes, intimes 🞏  Je ne comprends pas l’utilité 🞏  Autre 🞏  Si autre, préciser : _______________________________  Pas de réponse 🞏 |


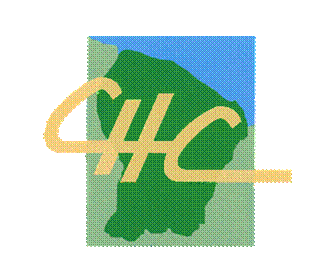

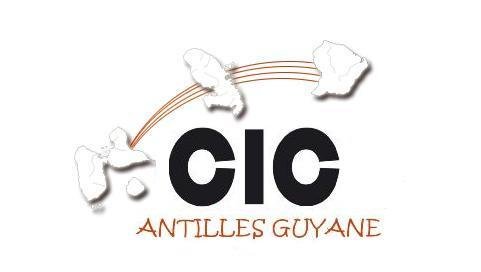


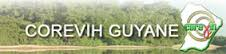


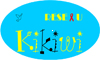


**
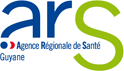
**

**Annexe XXXX : ccord d’engagement et de confidentialité des enquêteurs**

Titre de l’étude :

**Enquête connaissances attitudes et pratiques des détenus du centre pénitentiaire de Rémire-Montjoly vis-à-vis du VIH et des IST**

Titre abrégé :

**KABP prison**

Je soussigné(e) : _____________________________________________________

Coordonnées : _______________________________________________________

**______________________________________________________**

M’engage en ma qualité d’enquêteur en recherche épidémiologique, à :

Suivre la formation dispensée pour la conduite de cette étude

Respecter le calendrier de l’étude

Soumettre conformément à la formation, les items du questionnaire

Informer clairement le participant et s’assurer de son consentement à la participation

Respecter la confidentialité des données recueillies

Respecter l’anonymat des participants

Ne pas associer ni profiter de ma position pour entreprendre des démarches dans un but personnel, politique, religieux ou autre

J’accepte que mon nom soit cité dans les remerciements des communications orales et écrites publiées pendant ou à l’issue de l’étude

Oui ☺ Non ☹

Fait le _ _ /_ _/_ _ _ _ à

Prénom et Nom de l’enquêteur

Signature
