# Supplementary material for: Penile implants and other high risk practices in French Guiana’s correctional facility: A cause for concern
Source: PLoS One. 2019 Jun 28;14(6):e0218992. doi: 10.1371/journal.pone.0218992 (PMC6599133; doi:10.1371/journal.pone.0218992)
Supplement: S2 File — (DOCX) [file pone.0218992.s002.docx]

**Connaissances Attitudes Pratiques**

**des détenus face au VIH/SIDA et IST**

**N° de questionnaire :**

| \|__\|\|__\|\|____\|__\|__\|\|__\| |
| --- |

Numéro aléatoire séquentiel

**Date de l'entretien :**

|__||__||__||__||__||__|

**Introduction des enquêteurs**:

Hello, my name is.....I am a ….I’m working on a medical thesis, in association with Kikiwi group. We are studying which knowledge prisoners have about sexual diseases.Our goal is to improve the transmission of preventive messages in prisons.

If you agree to answer my questions, this questionnary takes about 50 minutes, it’s anonymous, and you’re free to refuse. This questionnary is proposed to a numerous number of prisoners, in order to understand better your world and identify health problems, and try to help you the best we can. Thank you for answering with sincerity to this questionnary.

**Consentement des personnesinterrogées :**

I accept to participate to this study and answer the questions. I understand that I ‘m free to refuse at any time, without any consequences, I agree that I’ll answer with sincerity to the questions.

**1-GENERAL CARACTERISTICS**

« I’ll ask you general questions about you»

| **N°** | **Questions** | **Catégories de codification** | **Aller à** |
| --- | --- | --- | --- |
| 1 | Sex of the respondent | Male: 1  Female: 2  Transexual : 3 |  |
| 2 | How old are you ? | Age (completed years) \|__\|\|__\|  I don’t know : 88  No response : 99 |  |
| 3 | In which country were you born ? | France : 1  Suriname : 2  Haïti : 3  Brésil : 4  Guyana : 5  Dominican Republic : 6  St Lucia : 7  Other : 8  If other, specify:…………  No response : 99 |  |
| 4 | How long have you been living in French Guyana? | Number of completed years \|__\| :1  Always : 2  I don’t know : 88  No response : 99 |  |
| 5 | What is our mother tongue? | French : 1  Dutch : 2  Bushinenguetongoe : 3  Portuguese (Brasil) : 4  English: 5  Spanish : 6  Haitian Creole : 7  Guyanese Creole : 8  Other :specify……………… : 9  No response : 99 |  |
| 6 | Do you know how to read and write ? | Yes, both : 1  Yes, only read : 2  Yes, only write : 3  No : 4  No response : 99 |  |
| 7 | Until what age were you in school? | Age in years\|__\|\|__\|  Ongoing studies : 1  I don’t kwow : 2  No response : 99 |  |
| 8 | At what level did you finish your studies? | CAP/BEP/BEPC : 1  Bachelor degree : 2  Graduate : 3  Without degree, stop before age 16 : 4  Without degree, stop after age 16 : 5  Others : specify………………..: 6  I never went to school : 7  I don’t know: 88  No response: 99 |  |
| 9 | Where did you live the month just before your incarceration? | Family home : 1  Personal house : 2  House, apartment for rent : 3  Roommate : 4  Public housing: 5  Room in a private : 6  Squatter (cabin, shed…): 7  Squat : 8  Homeless : 9  On the street: 10  Other: specify……….. : 11  No response: 99 |  |
| 10 | Which city and which neighbourhood did you live in the month before incarceration? | City :  Neighbourhood : |  |
| 11 | What was your situation just before incarceration? | Married : 1  Couple, living together: 2  Couple, not living together : 3  Single : 4  Widower : 5  Divorced : 6  No response: 99 |  |
| 12 | Do you have any children ? | Yes: 1  No : 2  No response : 99 |  |
| 13 | Before incarceration, what were your main incomes?  (multiple answers) | Incomes related to registered activity, please specify :……………………: 1  Incomes from jobs, fishing activity, farm products: 2  Family support : 3  Handicap support (AAH): 4  Unemployment allowance : 5  RSA : 6  Retirement incomes, minimum pension : 7  No incomes: 8  Begging, panhandle : 9  Sex work: 10  Stealing: 11  Other : specify…………… : 12  No response: 99 |  |
| 14 | Is religion important for you? | Very important : 1  Important : 2  Not very important: 3  Not important at all : 4  I don’t know: 88  No response: 99 |  |

**2-CARCERAL LIFE**

| 15 | In which unity do you reside? | -CD (detention center) : 1  -MA (remonded) : 2  -District women : 3 |  |
| --- | --- | --- | --- |
| 16 | Is this your first incarceration? | Yes : 1  No : 2  No response: 99 | **Aller à Q17** |
| 17 | If NO, how many times have youbeen incarcerated?  **Répondre si Q16=2** | Number of times: ____________  I don’t know : 88  No response: 99 |  |
| 18 | For this incarceration, how long have you been incarcerated ? | Number of months : _______  I don’t know : 88  No response : 99 |  |
| 19 | In your life, how long have you spent in total in prison ? | Number of months : _______  I don’t know : 88  No response : 99 |  |
| 20 | With how many people do you share your cell? | 0 : 1  1 : 2  2 : 3  3 : 4  4 : 5  5 : 6  No response : 99 |  |

« I’m going to ask you some questions about your living conditions in detention »

**3-INVENTORY of CONSUMPTIONS and PSYCHIATRIC COMOBIDITIES**

“I’m going to ask you some questions about your use of drugs and alcohol and psychiatric antecedents”

| 21 | **Before incarceration**, on which frequency were you taking alcohol ?  **Si Q21= 1 ne pas poser Q22,23,24** | Never : 1  Once per month : 2  2 to 4 times per month : 3  2 to 3 times by week : 4  4 times or more by week : 5  I don’t know : 88  No response : 99 |  |  |
| --- | --- | --- | --- | --- |
| 22 | **Before incarceration,** how many standard glasses (or cans) were you drinking during an ordinary day you drink alcohol ? | One or two : 1  Three or four : 2  Five or six : 3  Seven to nine : 4  Ten or more : 5 |  |  |
| 23 | **Before incarceration**,how many times were you drinking 6 standard glasses (or cans) or more during the same occasion ? | Never : 1  Less than one time per month : 2  One time per month : 3  One time per week : 4  Almost every day: 5  I don’t know : 88  No response : 99 |  |  |
| 24 | **Before incarceration,** which beverage were you drinking more often ? | Rum : 1  Beer : 2  Whisky : 3  Bita : 4  Other : specify : _____________ : 5  I don’t know : 88  No response : 99 |  |  |
| 25 | **Since incarceration**,how many times have you consumed alcohol ?  **Si Q25=1, ne pas poser Q26,27,28** | Never : 1  Once per month : 2  2 to 4 times per month : 3  2 to 3 times by week : 4  4 times or more by week : 5  I don’t know : 88  No response : 99 |  |  |
| 26 | **Since incarceration,** how many standard glasses (or cans) are you drinking during an ordinary day you drink alcohol? | One or two : 1  Three or four : 2  Five or six : 3  Seven to nine : 4  Ten or more : 5 |  |  |
| 27 | **Since incarceration,**How many times are you drinking 6 standard glasses (or cans) or more during a same occasion ? | Never : 1  Less than one time per month : 2  One time per month : 3  One time per week : 4  Almost every day: 5  I don’t know : 88  No response : 99 |  |  |
| 28 | **Since incarceration,**Which beverage are you drinking more often ? | Rum : 1  Beer : 2  Whisky : 3  Bita : 4  Other : precise : _____________ : 5  I don’t know : 88  No response : 99 |  |  |
| 29 | **If you were consuming drugs, before incarceration**,you were consumming…  Marijuana, kali | Every day: 1  At least once a week: 2  Less than once a week: 3  Never 4  I don’t know : 88  No response : 99 |  |  |
| 30 | Crack | Every day: 1  At least once a week: 2  Less than once a week: 3  Never 4  I don’t know : 88  No response : 99 |  |  |
| 31 | Cocaïne | Every day: 1  At least once a week: 2  Less than once a week: 3  Never 4  I don’t know : 88  No response : 99 |  |  |
| 32 | Blaka : (black joint : cannabis+crack) | Every day: 1  At least once a week: 2  Less than once a week: 3  Never 4  I don’t know : 88  No response : 99 |  |  |
| 33 | Other :  Specify : ……………….. | Every day: 1  At least once a week: 2  Less than once a week: 3  Never 4  I don’t know : 88  No response : 99 |  |  |
| 34 | **If you were consuming drugs,** since you have been arrested, have you consummed :  Marijuana, kali | Every day: 1  At least once a week: 2  Less than once a week: 3  Never 4  I don’t know : 88  No response : 99 |  | |
| 35 | Crack | Every day: 1  At least once a week: 2  Less than once a week: 3  Never 4  I don’t know : 88  No response : 99 |  | |
| 36 | Cocaïne | Every day: 1  At least once a week: 2  Less than once a week: 3  Never 4  I don’t know : 88  No response : 99 |  | |
| 37 | Blaka : (black joint : cannabis+crack) | Every day: 1  At least once a week: 2  Less than once a week: 3  Never 4  I don’t know : 88  No response : 99 |  | |
| 38 | Other : Precise……………. | Every day: 1  At least once a week: 2  Less than once a week: 3  Never 4  I don’t know : 88  No response : 99 |  | |
| 39 | Do you think that it would be easy or difficult to obtain cannabis within 24hours, if you wanted some ? | Impossible to obtain : 1  Very difficult to obtain : 2  Rather difficult to obtain : 3  Quite simple to obtain : 4  Very easy to obtain : 5  I don’t know : 88  No response : 99 |  | |
| 40 | Have you ever been hospitalized in a psychiatry department? | Yes : 1  No : 2  I don’t know : 88  No response : 99 |  | |
| 41 | Have you followed or taken treatment at the UFPI (psychiatric ward of the prison) | Yes : 1  No : 2  I don’t know : 88  No response : 99 |  | |

**4-CONNAISSANCES, ATTITUDES et OPINIONS SUR LE VIH**

| 42 | Among thesedifferent risks anddiseases, can you tell me ifyou fearfor yourself, not at all, no, not bad, a lot?  Cancer | Not at all : 1  Little : 2  A good few : 3  So much : 4  I don’t know : 88 |  |
| --- | --- | --- | --- |
| 43 | HIV/AIDS | Not at all : 1  Little : 2  A good few : 3  So much : 4  I don’t know : 88 |  |
| 44 | Dengue | Not at all : 1  Little : 2  A good few : 3  So much : 4  I don’t know : 88 |  |
| 45 | The pesticide risk | Not at all : 1  Little : 2  A good few : 3  So much : 4  I don’t know : 88 |  |
| 46 | Infertility(the fact of not being ableto have children) | Not at all : 1  Little : 2  A good few : 3  So much : 4  I don’t know : 88 |  |
| 47 | **Ask if women** : get pregnant without having planned | Not at all : 1  Little : 2  A good few : 3  So much : 4  I don’t know : 88 |  |
| 48 | **Ask if men**: that your partner pregnant without you planning it | Not at all : 1  Little : 2  A good few : 3  So much : 4  I don’t know : 88 |  |

| 1. ***KNOWLEDGES***  \| 49 \| Can you tell whether or not the AIDS virus can be transmitted in each of the following circumstances: During sex without a condom \| Yes : 1  No : 2  I don’t know : 88  No response : 99 \|  \| \| --- \| --- \| --- \| --- \| \| 50 \| During sex with condom \| Yes : 1  No : 2  I don’t know : 88  No response : 99 \|  \| \| 51 \| In public toilets \| Yes : 1  No : 2  I don’t know : 88  No response : 99 \|  \| \| 52 \| Drinking from the glass of an infected person \| Yes : 1  No : 2  I don’t know : 88  No response : 99 \|  \| \| 53 \| Bya mosquito bite \| Yes : 1  No : 2  I don’t know : 88  No response : 99 \|  \| \| 54 \| During a drug sting with a used syringe \| Yes : 1  No : 2  I don’t know : 88  No response : 99 \|  \| \| 55 \| Kissing an infected person \| Yes : 1  No : 2  I don’t know : 88  No response : 99 \|  \| \| 56 \| From mother to child during pregnancy or breastfeeding \| Yes : 1  No : 2  I don’t know : 88  No response : 99 \|  \| \| 57 \| I will quote a number of possible ways to protect yourself from AIDS. For each of them, do you think a very effective way, agree, disagree, or not at all effective?  Wash after sex \| Very effective: 1  Quite effective: 2  Ineffective: 3  Not at all effective : 4  I don’t know : 88  No response : 99 \|  \| \| 58 \| Have sex with few different partners \| Very effective: 1  Quite effective: 2  Ineffective: 3  Not at all effective : 4  I don’t know : 88  No response : 99 \|  \| \| 59 \| Use a male condom \| Very effective: 1  Quite effective: 2  Ineffective: 3  Not at all effective : 4  I don’t know : 88  No response : 99 \|  \| \| 60 \| Request AIDS virus screening test to partners \| Very effective: 1  Quite effective: 2  Ineffective: 3  Not at all effective : 4  I don’t know : 88  No response : 99 \|  \| \| 61 \| Do regularly a AIDS test \| Very effective: 1  Quite effective: 2  Ineffective: 3  Not at all effective : 4  I don’t know : 88  No response : 99 \|  \| \| 62 \| Withdraw before the end of sexual intercourse \| Very effective: 1  Quite effective: 2  Ineffective: 3  Not at all effective : 4  I don’t know : 88  No response : 99 \|  \| \| 63 \| Choose partnersthat are thoughtsafe \| Very effective: 1  Quite effective: 2  Ineffective: 3  Not at all effective : 4  I don’t know : 88  No response : 99 \|  \| \| 64 \| Anal penetration transmits less HIV than vaginal penetration \| True : 1  False : 2  I don’t know : 88  No response : 99 \|  \|   ***B.*ATTITUDES, STIGMA AGAINST PEOPLE LIVING WITH HIV**  « Let’s talk about what society, community, family, think about people with HIV »  If you knew that a person was infected with HIV, would you accept…:   \| 65 \| To work with this person ? \|  \| Yes : 1  No: 2  Perhaps : 77  I don’t know: 88  Pas de réponse : 99 \|  \| \| --- \| --- \| --- \| --- \| --- \| \| 66 \| To eat with this person? \|  \| Yes : 1  No: 2  Perhaps : 77  I don’t know: 88  Pas de réponse : 99 \|  \| \| 67 \| That this person cook with you? \|  \| Yes : 1  No: 2  Perhaps : 77  I don’t know: 88  Pas de réponse : 99 \|  \| \| 68 \| To share your cell with this person? \|  \| Yes : 1  No: 2  Perhaps : 77  I don’t know: 88  Pas de réponse : 99 \|  \| \| 69 \| To have sex using condoms? \|  \| Yes : 1  No: 2  Perhaps : 77  I don’t know: 88  Pas de réponse : 99 \|  \| | | | | |  |
| --- | --- | --- | --- | --- | --- | --- | --- | --- | --- | --- | --- | --- | --- | --- | --- | --- | --- | --- | --- | --- | --- | --- | --- | --- | --- | --- | --- | --- | --- | --- | --- | --- | --- | --- | --- | --- | --- | --- | --- | --- | --- | --- | --- | --- | --- | --- | --- | --- | --- | --- | --- | --- | --- | --- | --- | --- | --- | --- | --- | --- | --- | --- | --- | --- | --- | --- | --- | --- | --- | --- | --- | --- | --- | --- | --- | --- | --- | --- | --- | --- | --- | --- | --- | --- | --- | --- | --- | --- | --- | --- | --- | --- | --- | --- |
| 1. **OPINIONS ABOUT MALE CONDOMS**   « I’ll ask you some questions about male condoms» | | | | | |
| 70 | Do you feel concerned by information campaigns on HIV/AIDS? |  | Not at all concerned : 1  Slightly concerned: 2  Very concerned : 3  I don’t know : 88  No response: 99 |  | |
| 71 | I will ask your opinion on a number of discussion topics affecting society. For each one, can you tell me if you totally agree, somewhat agree, tend to disagree, do not agree?  We must prevent a child with AIDS virus from going to school: |  | Totally agree : 1  Somewhat agree: 2  Tend to disagree : 3  No agree: 4  I don’t know : 88  No response : 99 |  | |
| 72 | Homosexuals are people like everyone else: |  | Totally agree : 1  Somewhat agree: 2  Tend to disagree : 3  No agree: 4  I don’t know : 88  No response : 99 |  | |
| 73 | Must isolate AIDS patients from the rest of the population |  | Totally agree : 1  Somewhat agree: 2  Tend to disagree : 3  No agree: 4  I don’t know : 88  No response : 99 |  | |
| 74 | Must develop sex education programs in schools |  | Totally agree : 1  Somewhat agree: 2  Tend to disagree : 3  No agree: 4  I don’t know : 88  No response : 99 |  | |
| 75 | AIDS is a punishment from God, a curse |  | Totally agree : 1  Somewhat agree: 2  Tend to disagree : 3  No agree: 4  I don’t know : 88  No response : 99 |  | |

« I’ll ask you some questions about male condoms»

|  | For each of thefollowing opinionsabout condoms, can you tell me ifyou agreeor disagree | |  |
| --- | --- | --- | --- |
| 76 | Condoms is for young people | Totally agree : 1  Somewhat agree: 2  Tend to disagree : 3  No agree: 4  I don’t know : 88  No response : 99 |  |
| 77 | Condom allows having sexwithout asking anyquestions | Totally agree : 1  Somewhat agree: 2  Tend to disagree : 3  No agree: 4  I don’t know : 88  No response : 99 |  |
| 78 | Condom is complicated to use with the same partner for a long time | Totally agree : 1  Somewhat agree: 2  Tend to disagree : 3  No agree: 4  I don’t know : 88  No response : 99 |  |
| 79 | Condoms create doubts on your partners | Totally agree : 1  Somewhat agree: 2  Tend to disagree : 3  No agree: 4  I don’t know : 88  No response : 99 |  |
| 80 | Using condom is something common | Totally agree : 1  Somewhat agree: 2  Tend to disagree : 3  No agree: 4  I don’t know : 88  No response : 99 |  |
| 81 | Condom is difficult to use systematically | Totally agree : 1  Somewhat agree: 2  Tend to disagree : 3  No agree: 4  I don’t know : 88  No response : 99 |  |
| 82 | Is it easy to use condoms in prison? | Yes: 1  No : 2  I don’t know : 88  No response : 99 | **Si non aller à Q83** |
| 83 | If NO, why ?  **Si Q82=1 , ne pas poser Q83** | There is no condoms : 1  I’m ashamed to take condoms: 2  I don’t know where they are : 3  They are notplacedin the right place: 4  Other, specify………………… : 5  I don’t know: 88  No response : 99 |  |

***D.* TREATMENTS of HIVand AIDS**

« We will talk about treatments available»

| 84 | Have you ever heard about a treatment for HIV/AIDS? | Yes : 1  No : 2  I don’t know : 88  No response : 99 |  |
| --- | --- | --- | --- |
| 85 | With these treatments, HIV-positive people can live normally? | Yes : 1  No : 2  I don’t know : 88  No response : 99 |  |
| 86 | With these treatments, it definitely cure AIDS | Yes : 1  No : 2  I don’t know : 88  No response : 99 |  |
| 87 | People with HIV who take proper treatment less transmit HIV | Yes : 1  No : 2  I don’t know : 88  No response : 99 |  |
| 88 | Have you heard of the post-exposure treatment (an emergency treatment) which, taken just after unprotected intercourse, may reduce the risk of being infected with the AIDS virus? | Yes : 1  No : 2  I don’t know : 88  No response : 99 |  |
| 89 | Have you everheard of circumcise?  (ablation of the foreskin)  **Si Q89=2 expliquer ce qu’est la circoncision** | Yes : 1  No : 2  I don’t know : 88  No response : 99 |  |
| 90 | Circumcise reduces by 60% the risk of infection for HIV, would it be acceptable for you if you were asked to do it? | Yes : 1  No : 2  I don’t know : 88  No response : 99 |  |

**E.PERCEIVED RISK OF BECOMING INFECTED WITH HIV**

“We'll now talk about the risk of becoming infected with HIV”

| 91 | Do you consider that compared to an average person, you have morerisk, thesame risk,less riskor no riskof becoming infectedwith the AIDSvirus? | More risk: 1  The same risk : 2  Less risk : 3  No risk : 4  I don’t know : 88  No response : 99 |  |
| --- | --- | --- | --- |
| 92 | Yourself, have you ever feared to have been contaminated with the AIDS virus? | Yes, several times : 1  Yes, one time : 2  No : 3  I don’t know : 88  No response : 99 |  |

**5-SEXUAL HISTORY**

« I will now ask you some questions about your sexual relationships; our conversation will remain strictly confidential »

***A. FIRST SEX***

**«**Let's talk first sex you've had in your life**»**

| 93 | How old were you when you first had sex? | |  | | Age in years [__\|__]  I don’t know : 88  No response : 99  I never had sex : 77 |  |
| --- | --- | --- | --- | --- | --- | --- |
| 94 | This first sex was something: | |  | | Whether you wantat this time: 1  You acceptbut donot want really : 2  You were forced to do against your will: 3  No response : 99 |  |
| 95 | During this first sex, did you use a condom? | |  | | Yes : 1  No : 2  I don’t know : 88  No response : 99 |  |
| 96 | How many different sex partners have you had in your life ? |  | | 5 and less : 1  10 and less : 2  20 and less : 3  between 20 and 40 : 4  More than 40 : 5  I don’t know: 88  No response: 99 | |  |
| 97 | How many different sex partners have you had during the last 12 months ? |  | | Total ____  I don’t know : 88  No response : 99 | |  |
| 98 | **Ask if men**  In your life, Have you had sexual intercourse… |  | | Only with women : 1  With men and women : 2  Only with men : 3  No response : 99 | |  |
| 99 | **Ask if women**  In your life, Have you had sexual intercourse… |  | | Only with men : 1  With men and women : 2  Only with women : 3  No response : 99 | |  |
| 100 | During the last 5 years, have you had several partners during the same period, ie begin to have sexual intercourse with a person, continuing having sexual intercourse with another? |  | | Yes : 1  No : 2  No response : 99 | |  |
| 101 | And during the last 12 months, have you had several partner during the same period, ie beginning having sexual intercourse with a person continuing having sexual with another? |  | | Yes : 1  No : 2  No response : 99 | |  |
| 102 | During the last five years, have you have had, at least one time, sexual intercourse by paying or getting payed? |  | | Yes, paying : 1  Yes, getting paid : 2  No : 3  No response : 99 | |  |
| 103 | During the last 12 months, have you have had, at least one time, sexual intercourse by paying or getting payed?  **Si Q103=3 , ne pas poser Q104** |  | | Yes, paying : 1  Yes, getting paid : 2  No : 3  No response : 99 | |  |
| 104 | Did you use a condom? |  | | Yes : 1  No : 2  No response : 99 | |  |

***B. SEXUAL LIFE IN PRISON***

“We willask you some questionsabout your sex lifein prison”

| 105 | Have you had sex since your incarceration?  **SI Q105=2 ne pas poser Q106, Q107,Q108** | Yes : 1  No : 2  No response : 99 | Q106 |
| --- | --- | --- | --- |
| 106 | This last sexual intercourse was : | With a woman : 1  With a man : 2  No response : 99 |  |
| 107 | Where did you have sex?  (plusieurs réponses possibles) | At parlor : 1  In jail (showers, cell..) : 2  Other, specify………………… : 3  No response : 99 |  |
| 108 | Did you use a condom during this last sex? | Yes: 1  No : 2  No response: 99 |  |
| 109 | Beforedetention, did anybody forceyouto havesex? | Yes: 1  No : 2  No response: 99 |  |
| 110 | In detention, did anybody forceyouto havesex? | Yes: 1  No : 2  No response: 99 |  |

**6-PENILE IMPLANTSand BLOODY PRACTICES**

**Ne pas poser Q111 à Q122 si femme**

| 111 | Did you have inserted or implanted an object under the skin of your penis ?(dominos)  **Si Q111=2 ne pas poser Q112 à 122** | Yes : 1  No : 2  No response : 99 | **Q112** |
| --- | --- | --- | --- |
| 112 | If yes, how many? (si 1 à la Q111) | Number \|_ _\|_ _\| | **Q113** |
| 113 | Did you do that, while you were in prison? | Yes, all : 1  Yes, not all : 2  No, anyone : 3  No response : 99 |  |
| 114 | Did the operation was performed : | Alone : 1  With a help of somebody : 2  Pas de réponse : 99 |  |
| 115 | Installation was performed : | For free : 1  With a remuneration : 2  No response : 99 |  |
| 116 | What was the material used for the installation? | Razor blade : 1  Can cover:2  Other , specify………………. : 3  No response: 99 |  |
| 117 | The material was :  (deux réponses possibles) | New : 1  Used : 2  Disinfected : 3  Soiled (not disinfected) : 4  No response : 99 |  |
| 118 | Were there any complications after putting it on you?  **Si Q118=2 ne pas poser Q119** | Yes : 1  No : 2  I don’t know : 88  No response : 99 | **Q119** |
| 119 | If so, whichones?  **SI Q118=22 ne pas poser Q119** | Swelling : 1  Pain : 2  Fever: 3  Non stop bleeding : 4  Erection problems : 5  Healing problems : 6  Other, specify…………… : 7  No response : 99 |  |
| 120 | Why do you put bouglous?  (do not quote the suggestions)) | Because I findit beautiful : 1  To increase sexual pleasure of my partner : 2  To increase my sexual pleasure: 3  To make sex painful : 4  To have a girl easily : 5  Because I’m part of a clan:6  Other : specify…………… : 7  I don’t know : 88  No response : 99 |  |
| 121 | Do you think that bouglous are an inconvenience for the use of condom ?(difficult to put, condom breaking) | Yes : 1  No : 2  I don’t know : 88  No response : 99 |  |
| 122 | Have you ever used two condoms on each other during sex? | Yes : 1  No : 2  I don’t know : 88  No response : 99 |  |
| 123 | Did you get a tattoo or a piercing during your incarceration? | Yes : 1  No : 2  I don’t know : 88  No response : 99 |  |

**7-SEXUALLY TRANSMITTED INFECTIONS**

“We will now talk about the diseases that can be transmitted during sex”

| 124 | How many times in your life, did you get an infection or disease that can be sexually transmitted during sex? | Number of times______  Never : 1  I don’t know : 88 |  |
| --- | --- | --- | --- |
| 125 | We will talk about this last time, when was the disease? | Less than one year : 1  More than one year but less than five years : 2  5 years and more : 3  No response : 99 |  |
| 126 | What disease or infection was it (last time)?  (Quote if necesary) | Mycosis/mushrooms / candida: 1  Chlamydia : 2  Gonococcal /Gonorrhea /  clap: 3  Trichomonas : 4  Syphilis : 5  Papillomavirus /warts / cockscomb : 6  Hepatitis B : 7  Mycoplasma : 8  Genital herpes : 9  HIV / AIDS : 10  Other: specify ……………  : 11  I don’t know the name : 12  No response : 99 |  |
| 127 | Have you warned your partner that you had this disease or infection? (consigne enquêteur : citer) | Yes : 1  Yes but not for all : 2  No : 3  It was him or them who warned me : 4  No response : 99 |  |

**8-HIV TESTING**

« We'lltalk about HIVscreening test»

| 128 | Have you already made a blood test for HIV?  (consigne enquêteur : si « Oui », relancer « Une fois ? Plusieurs fois ? ») | Yes, several times : 1  Yes, one time : 2  No : 3  I don’t know : 88  No response: 99 | **Q132**  **Q132** |
| --- | --- | --- | --- |
| 129 | Did you made this test during the last five years?  (consigne enquêteur : if yes, ask : « one time, several times ?») | Yes, several times : 1  Yes, one time : 2  No : 3  I don’t know : 88  No response: 99 | **Q132**  **Q132** |
| 130 | And during the last 12 months? (consigne enquêteur : si « Oui », relancer « Une fois ? Plusieurs fois ? » | Yes, several times : 1  Yes, one time : 2  No : 3  I don’t know : 88  No response: 99 | **Q132**  **Q132** |
| 131 | About your serological status, you would say that… | You are negative  (you’re not infected with the AIDS virus) : 1  You were HIV-negative at the last test but you are not sure of the yet to be now: 2  You are positive (You’re contaminated with the AIDS virus): 3  No response : 99 |  |
| 132 | He you made a blood test for HIV since your are in prison ? | Yes : 1  No : 2  I don’t know : 88  No response : 99 | **Q 134** |
| 133 | Were you asked to come get the results of this test?  **Si Q132=1** | Yes : 1  No : 2  I don’t know : 88  No response : 99 |  |
| 134 | Do you know your serological status with respect to hepatitis C? | Yes : 1  No : 2  I don’t know : 88  No response : 99 | **Q135** |
| 135 | If yes, what is it?  **Poser si Q134=1** | Negative: 1  Positive : 2  I don’t know : 88  No response : 99 |  |

**9-PROXIMITE A LA MALADIE ET AUX PERSONNES SEROPOSITIVES**

| 136 | Do you know personally, in your environment (family, friends, colleagues ...) one or more person with HIV or AIDS?(consigne enquêteur : citer)  **Si Q137=3 ne pas poser Q138,Q139,Q140** | Yes, one person : 1  Yes, several persons : 2  No, anyone : 3  I don’t know : 88  No response : 99 | **Q 137**  **Q137** |
| --- | --- | --- | --- |
| 137 | Is it…  (consigne enquêteur : si plusieurs personnes connues – relancer «Or any other person?»)    **Poser si Q136=1 ou 2** | Your currentsexual partnerorone of yourcurrentpartners: 1  A parent : 2  A friend : 3  A person with whom you had sex in the past: 4  A colleague : 5  A knowledge : 6  Someone you've heard without knowing personally: 7  Yourself : 8  Other : specify_______________ : 9  No response : 99 |  |
| 138 | Are you aware of HIV or AIDS patients in the prison? | Yes : 1  No : 2  No response : 99 | **Q139** |
| 139 | How do you know it / how do you believe it??  **Poser si Q138=1** | Because it shows: 1  Because the person told me about her HIV status: 2  Because someone talled me: 3  Others: specify :……….. : 88 |  |

**10-PREVENTION INTERVENTION and MEDIAS USE**

“We'll talk about HIV messages that you may have in the media”

| 140 | Have you ever read, seen or heard about HIV/AIDS?  *(plusieurs réponses possibles)* | In newspaper : 1  On television : 2  On radio : 3  On posters in the street : 4  Groups, associations: 5  Never : 6  I don’t know : 88  No response : 99 |  |
| --- | --- | --- | --- |
| 141 | Last month, you would rather say that  -You listened tothe radio: | Every day : 1  At least once a week : 2  Less than once per week : 3  Never : 4  I don’t know : 88  No response :99 |  |
| 142 | -You watched TV : | Every day : 1  At least once a week : 2  Less than once per week : 3  Never : 4  I don’t know : 88  No response :99 |  |
| 143 | Whatradio stationsdo you listen to?  *(plusieurs réponses possibles)* | Guyane Première : 1  Radio Gabriel : 2  NRJ : 3  KFM : 4  Mosaïk : 5  Trace FM : 6  Other………………… : 7  I don’t know : 88  No response : 99 |  |
| 144 | What TV channels do you watch?  *(plusieurs réponses possibles)* | Guyane Première : 1  Tempo : 2  France O : 3  Other, precise..................…. 4  I don’t know : 88  No response : 99 |  |
| 145 | On what topics you would like to have more information? | On HIV transmission modes: 1  About the screening test of HIV : 2  On treatments against HIV : 3  On condoms : 4  Other : specify………………: 5  I don’t know : 88  No response : 99 |  |
| 146 | What other health issues or any other subject would you be willing to get information? | Free answers :  I don’t know : 88  No response : 99 |  |
